# Cost‐Effective H_2_O_2_‐Regeneration of Powdered Activated Carbon by Isolated Fe Sites

**DOI:** 10.1002/advs.202204079

**Published:** 2022-11-18

**Authors:** Xu Chen, Ziqi Tian, Qihao Yang, Linjuan Zhang, Qiu Yang, Liang Chen, Zhiyi Lu

**Affiliations:** ^1^ Key Laboratory of Advanced Fuel Cells and Electrolyzers Technology of Zhejiang Province Qianwan Institute of CNITECH Ningbo Institute of Materials Technology and Engineering Chinese Academy of Sciences Ningbo Zhejiang 315201 P. R. China; ^2^ University of Chinese Academy of Sciences Beijing 100049 P. R. China; ^3^ Key Laboratory of Interfacial Physics and Technology Shanghai Institute of Applied Physics Chinese Academy of Sciences Shanghai 201800 P. R. China; ^4^ Ningbo New Material Testing and Evaluation Center Co., Ltd Ningbo New Materials Innovation Center Ningbo Zhejiang 315201 P. R. China

**Keywords:** Fe‐based reactive oxygen species (ROS), nonradical Fenton‐like, powdered activated carbon, regeneration

## Abstract

The reuse of powdered activated carbon (PAC) vitally determines the economics and security of the PAC‐based adsorption process, while state‐of‐the‐art PAC regeneration technologies are usually unsatisfactory. Here, it is demonstrated that isolated Fe sites anchored on commercial PAC enable fast H_2_O_2_ activation to produce Fe‐based reactive oxygen species for highly efficient PAC regeneration at room temperature. Taking rhodamine B as a representative pollutant, PAC decorated with isolated Fe sites realize H_2_O_2_ based regeneration with negligible adsorption capacity degradation for 10 cycles. Moreover, in terms of the PAC loss rate, this technology is greatly superior to traditional Fenton‐based regeneration technology. Further operando experiments and theoretical calculations reveal that the high regeneration performance can be attributed to the isolated HO—Fe=O motifs, which activate H_2_O_2_ via a nonradical reaction pathway. These findings provide a very promising strategy toward reducing the cost of H_2_O_2_‐based PAC regeneration technology.

## Graphical abstract

Commercial powdered activated carbon decorated with isolated Fe‐O_5_ sites enables fast H_2_O_2_ activation and realizes H_2_O_2_‐based regeneration with negligible adsorption capacity degradation for ten cycles. Further operando experiments and theoretical calculations reveal that the high regeneration performance is attributed to the isolated HO—Fe=O motifs, which activate H_2_O_2_ via a nonradical reaction pathway to produce Fe‐base ROS.

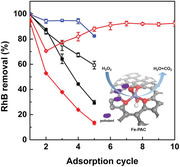

## 1 Introduction

Activated carbon (AC) is irreplaceable and widely adopted in drinking water purification and wastewater treatment.^[^ ^1^ ^]^ Its unique advantages, including its high specific surface area, nontoxicity, and acid/base resistance, determine AC as an outstanding adsorbent for removing organic pollutants from water.^[^ ^1^, ^2^ ^]^ Compared with granular activated carbon (GAC), powdered activated carbon (PAC) enables an accelerated mass transfer process, thus enhancing the adsorption kinetics.^[^ ^3^ ^]^ However, once AC has been used in adsorbing toxic and nonbiodegradable organic pollutants for a specific period of time, the limited absorption capability and toxicity prevent its reuse.^[^ ^2^, ^4^ ^]^ Hence, in terms of not only economy but also security, sustainable, and cost‐effective AC regeneration methods post treatment are desperately needed.^[^ ^1^, ^2^, ^5^ ^]^

Industrially, the major state‐of‐the‐art AC regeneration technology is thermal regeneration,^[^ ^2^, ^6^ ^]^ which involves processes of drying, thermal desorption, pyrolysis, carbonization under an inert atmosphere and gasification of pyrolytic residue in a mildly oxidizing atmosphere (H_2_O, CO_2_, or air).^[^ ^2^, ^6^, ^7^ ^]^ Because of the complex engineering process and the high operation temperature (typically 700–1 000 °C), thermal regeneration technology is highly energy consumptive and relies heavily on initial equipment investment.^[^ ^4^, ^6^, ^8^ ^]^ More importantly, due to the possibility of dust explosion, it is extremely prudent to employ thermal regeneration technology for PAC regeneration.

To circumvent the restrictions of thermal regeneration technology, H_2_O_2_‐based Fenton regeneration technology, which utilizes in situ generated reactive oxygen species (ROS) from additional H_2_O_2_ to almost completely mineralize absorbed organic pollutant, has been demonstrated as a simple and low investment AC (including PAC) regeneration technology.^[^ ^2^, ^9^ ^]^ The representative ROS, hydroxyl radical (·OH), possesses strong oxidizing ability (≈2.8 V) and hence enables the oxidation of most organic pollutants.^[^ ^2^, ^10^ ^]^ However, for the traditional homogeneous Fenton regeneration, the solution pH should be adjusted to 2–4 and sacrifice of Fe^2+^ is necessary.^[^ ^11^ ^]^ More noteworthy, the AC would inevitably be lost during the regeneration because of nonselective attacks by free radicals, resulting in fairly expensive cost ($1.32 kg^−1^ or even higher for GAC regeneration).^[^ ^9a^ ^]^ It should be noted that, due to the much larger adsorption capacity of PAC, the H_2_O_2_ cost for PAC regeneration is apparently higher than that for GAC regeneration. In this regard, developing a simple process and simultaneously improving the regeneration efficiency becomes key for the large‐scale application of this technology.

In this work, it was discovered that constructing isolated Fe sites anchored on commercial PAC (Fe‐PAC) can efficiently utilize H_2_O_2_ and realize a low cost and simple regeneration process. Taking Rhodamine B (RhB) as a representative pollutant, the absorption capability of Fe‐PAC could be regenerated over 10 cycles with an efficiency of 70.5–92.7% within 24 h, and the regeneration cost was as low as ≈$0.35 kg^−1^. Similar results were obtained in simulated wastewater containing other model contaminants (methylene blue and crystal violet). Compared with homogeneous Fenton regeneration, this process is not only simple (in a neutral system without addition of Fe^2+^), but also allows for a significant reduction of activated carbon losses (the average loss is less than half of that in the homogeneous process). Detailed operando experiments and density functional theory (DFT) calculations both revealed that, instead of the aqueous phase radical pathway in traditional H_2_O_2_‐based regeneration, the isolated HO—Fe=O motifs in Fe‐PAC effectively activate H_2_O_2_ to nonradical ROS, which enables a selective attack on the pollutants adsorbed on the PAC surface. This work for the first time demonstrates the vital role of atomically dispersed sites in PAC regeneration, and at the same time provides a theoretical and material basis for prolonged large scale H_2_O_2_‐based regeneration technology.

## 2 Results

### 2.1 H_2_O_2_‐Regeneration Process of Fe‐PAC

**Figure 1:**
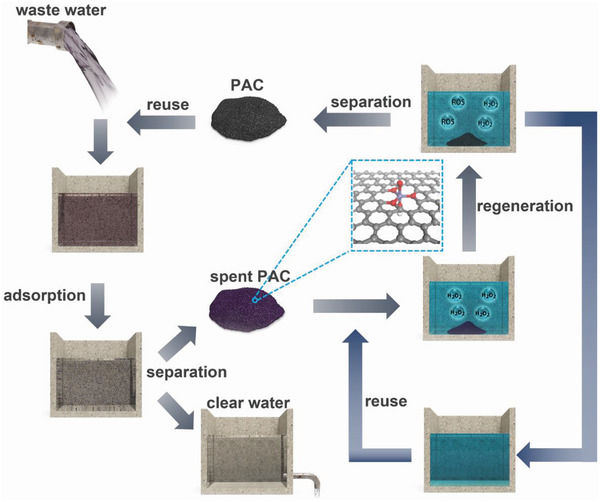
Schematic illustration of the H_2_O_2_‐based PAC adsorption/regeneration process for wastewater treatment.

The traditional H_2_O_2_‐based regeneration process of activated carbon is briefly explained in **Figure** **1**. It involves three simple processes: i) separation of spent PAC post wastewater treatment; ii) regeneration of spent PAC in H_2_O_2_ solution through Fenton reaction; iii) reuse of the regenerated PAC in the next adsorption/regeneration cycle. However, in homogeneous Fenton regeneration, the radicals generated by reaction with Fe^2+^ in the aqueous phase would nonselectively attack the pollutant and PAC, giving rise to severe PAC loss and low utilization of H_2_O_2_. Therefore, it was hypothesized that, if the active Fe sites can be anchored on the PAC surface with an appropriate coordination environment, the distance from the generated ROS to the organic pollutant would be drastically shortened, resulting in preferential attack toward the adsorbed organic pollutants.^[^ ^12^ ^]^

**Figure 2:**
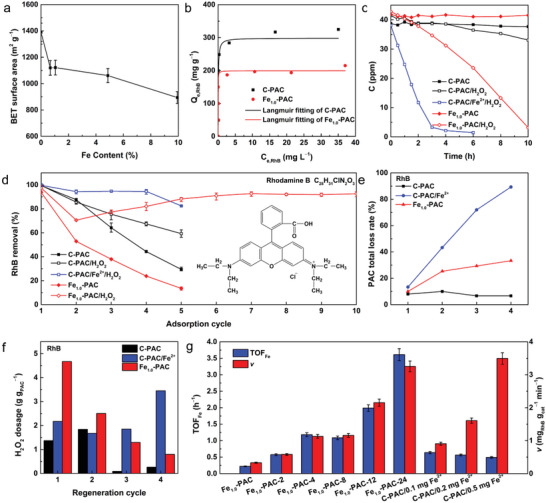
a) The BET surface area of the as‐synthesized Fe‐PAC with different Fe contents. b) The adsorption equilibrium isotherms of C‐PAC and Fe_1.0_‐PAC at 25 °C for 24 h; data are fitted using the Langmuir isotherm model. c) Comparison of RhB degradation rate in different system. [C‐PAC] = [C‐PAC/Fe^2+^] = [Fe_1.0_‐PAC] = 0.2 g L^−1^, [H_2_O_2_] = 600 mmol. d) Comparison of cyclic RhB removal performance in different systems. [RhB] = 20 ppm (≈50% of Fe_1.0_‐PAC saturation adsorption), adsorption time = 30 min. e) Comparison of PAC loss during regeneration process in different systems. f) Comparison of H_2_O_2_ dosage during regeneration process in different system. g) Comparison of RhB degradation rate (*v*) and TOF_Fe_ of Fe_1.0_‐PAC with different activation times and C‐PAC/Fe^2+^ with different Fe^2+^ contents.

To verify our hypothesis, a series of Fe‐PAC with tunable Fe contents (0.5–10%) was synthesized via a simple pyrolysis strategy (see the Experimental Section for details). High‐resolution transmission electron microscopy images (HRTEM, Figure S1 in the Supporting Information) and X‐ray diffraction patterns (XRD, Figure S2 in the Supporting Information) both demonstrated that the Fe‐PAC with low Fe content (≤1 wt%) exhibited no observable nanoparticles, while further increasing the Fe content led to the formation of Fe‐based nanoparticles. The Brunauer‐Emmett‐Teller (BET) surface area decreased with increasing Fe content (**Figure** **2**a; and Figure S3 in the Supporting Information), which might be caused by the occupation of the pores of PAC by nanoparticles. Considering that the Fe‐PAC should simultaneously possess both high adsorption capacity and catalytic activity for H_2_O_2_, it is believed by this group that Fe‐PAC (≈1.0 wt%, Fe_1.0_‐PAC) with a relatively high surface area (≈1 120 m^2^ g^−1^) is desirable for both adsorption and H_2_O_2_‐regeneration. Further characterizations, such as aberration‐corrected high‐angle annular dark‐field scanning transmission electron microscopy (AC‐HAADF‐STEM, Figure S4 in the Supporting Information), Fourier Transform‐extended X‐ray absorption fine structure (FT‐EXAFS) and wavelet transform (WT), demonstrated that atomically dispersed Fe sites and Fe clusters coexisted in Fe_1.0_‐PAC (Figure S5a,b in the Supporting Information).

Prior to the regeneration process, the adsorption equilibrium isotherms (Figure 2b) and adsorption kinetics (Figure S7 in the Supporting Information) for RhB on Fe_1.0_‐PAC and commercial PAC (C‐PAC) were evaluated. It was observed that the maximum adsorption capacity of Fe‐PAC (*q*_m_) was ≈200 mg g^−1^, two‐thirds that of C‐PAC (≈300 mg g^−1^) by the Langmuir isotherm model. This phenomenon was attributed to the inevitable decrease of surface area after Fe modification. A catalytic RhB degradation test by H_2_O_2_ was then conducted (Figure 2c), where it was found that the RhB degradation rate in the Fe_1.0_‐PAC/H_2_O_2_ system (3.96 ppm h^−1^) was higher than that in the C‐PAC/H_2_O_2_ system (0.83 ppm h^−1^), while the addition of Fe^2+^ into the C‐PAC/H_2_O_2_ system resulted in a dramatically increased degradation rate (11.61 ppm h^−1^). These results can be easily understood by the much faster reaction rate of a homogeneous Fenton reaction than that of a heterogeneous Fenton‐like reaction.^[^ ^13^ ^]^ It should be noted that RhB could hardly be degraded without H_2_O_2_. These results directly revealed that H_2_O_2_ and Fe sites are indispensable for RhB degradation.

After the cyclic adsorption‐regeneration experiment, PAC loss measurements and H_2_O_2_ dosage calculations were performed to systematically evaluate the regeneration performance of Fe_1.0_‐PAC/H_2_O_2_, C‐PAC/H_2_O_2_, and C‐PAC/H_2_O_2_/Fe^2+^. As shown in Figure 2d, the removal efficiency of RhB on the Fe_1.0_‐PAC/H_2_O_2_ system was reduced from 97.5% (first adsorption) to 70.5% after the first regeneration cycle, and then gradually increased to 92.6% after the following eight cycles. In sharp contrast, without regeneration, the removal efficiency of Fe_1.0_‐PAC decreased to 13.5% after five adsorption attempts. The average absorbent loss (Figure 2e) and H_2_O_2_ consumption (Figure 2f) were 8.25% and 2.31 g g_(PAC)_^−1^ for Fe_1.0_‐PAC in four regeneration cycles (corresponding to five adsorption cycles). Assuming that the price of commercial 27.5 wt% H_2_O_2_ is $120/t, the regeneration costs are $2.04, $1.09, $0.56, and $0.35 per kg PAC for the 1st, 2nd, 3rd, and 4th cycles, respectively. The average cost ($1.01 kg_(PAC)_^−1^) is even lower than that reported for H_2_O_2_‐regeneration of GAC associated by additive Fe^2+^ ($1.32 kg_(GAC)_^−1^). It is interesting that the consumed H_2_O_2_ in the 1st cycle was apparently higher than in the following three cycles, possibly due to the additional activation of Fe_1.0_‐PAC besides pollutant degradation (discussed later). The Fe leaching rates were measured by Inductively Coupled Plasma (ICP) (Figure S9 in the Supporting Information), where it was observed that, although the Fe would leach out in the first cycle, the leaching rate decreased sharply in the following cycles. Moreover, the Fe sites were still atomically dispersed in the Fe_1.0_‐PAC after regeneration, proven by FT‐EXAFS and WT (Figure S5c,d in the Supporting Information). As to C‐PAC, although it could efficiently remove RhB in the first two adsorption cycles due to the high adsorption capacity (Figure 2d), it could hardly be regenerated in H_2_O_2_ solution, which was further confirmed by the low loss rate (6.67%, Figure 2e) and average H_2_O_2_ consumption (0.890 g g_(PAC)_^−1^, Figure 2f). The improved removal efficiency of C‐PAC after H_2_O_2_ solution treatment was attributed to desorption of RhB (Figure S10 in the Supporting Information). The introduction of Fe^2+^ into the regeneration progress could effectively restore the adsorption capacity (82.5–99.6% in five adsorption cycles, Figure 2d), while the loss rate reached an unacceptable value of 89.3% after four regeneration cycles (Figure 2e). Although the average H_2_O_2_ consumption was closer to the Fe_1.0_‐PAC system (2.29 g g_(PAC)_^−1^, Figure 2f), the consumed amounts of H_2_O_2_ in the 3rd and 4th cycles were much higher, indicating insufficient utilization of H_2_O_2_. Moreover, the Fe_1.0_‐PAC based adsorption‐regeneration process could be extended to other simulated wastewater, such as wastewater containing methylene blue (Figure S11 in the Supporting Information) and crystal violet (Figure S12 in the Supporting Information), demonstrating its general applicability.

The aforementioned results clearly demonstrated that Fe_1.0_‐PAC exhibited much better regeneration performance (i.e., catalytic activation of H_2_O_2_) after the 1st regeneration cycle, corresponding to an immersion time of 24 h in H_2_O_2_ solution. To examine this activation process, the Fe_1.0_‐PAC samples after immersion for different times (up to 24 h) were then employed as catalysts for Fenton‐like RhB degradation (see the Experimental Section for details). The turnover frequency of Fe sites (TOF_Fe_) in Fe_1.0_‐PAC was 0.22 h^−1^ initially and dramatically increased to 3.61 h^−1^ after 24 h activation (Figure 2g; and Table S1 in the Supporting Information). Due to the high TOF_Fe_ and well‐preserved loading mass of Fe, the reaction rate (*v*) and pseudofirst‐order kinetic (*k*) of Fe_1.0_‐PAC‐24 were 3.25 mg g^−1^ min^−1^ and 0.0466 min^−1^, respectively, ≈3.5‐fold and ≈4.5‐fold of those of C‐PAC with 0.1 mg Fe^2+^ system (0.907 mg g^−1^ min^−1^ and 0.0100 min^−1^; Figures S13 and S14 and Table S1 in the Supporting Information). Moreover, Fe_1.0_‐PAC‐24 outperformed Fe‐based catalysts reported in the literature with regards to H_2_O_2_ activation performance (Figure S15 and Table S2 in the Supporting Information).^[^ ^14^ ^]^

### 2.2 Characterization of Fe_1.0_‐PAC after H_2_O_2_ Treatment

**Figure 3:**
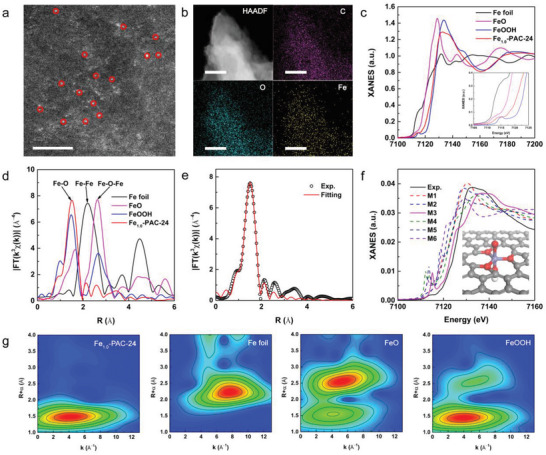
a) AC‐HAADF‐STEM image of Fe_1.0_‐PAC‐24 (scale bar 2 nm). b) HAADF‐STEM image and the corresponding EDS mapping images of Fe_1.0_‐PAC‐24 (C, purple; O, green; Fe, yellow; scale bar 200 nm). c) XANES spectra at the Fe K‐edge and pre‐edge region (inset) of the Fe_1.0_‐PAC‐24, Fe foil, FeOOH, and FeO. d) FT *k* ^3^‐weighted *χ*(*k*)‐function of the EXAFS spectra. e) FT magnitude of Fe K‐edge EXAFS spectra in R space for Fe_1.0_‐PAC‐24. Experimental (black line) and simulated (red circle). f) Comparison between the experimental K‐edge XANES spectra and the simulated spectra of six possible models of Fe_1.0_‐PAC‐24. g) WT of the Fe K‐edge.

A variety of characterization methods were adapted to trace the source of the high Fenton‐like performance of Fe_1.0_‐PAC‐24. Firstly, AC‐HAADF‐STEM results demonstrated that, different to Fe_1.0_‐PAC, the doped Fe was atomically dispersed in Fe_1.0_‐PAC‐24 nanoparticles after H_2_O_2_ activation, as shown in the circled dots in **Figure** **3**a. The energy‐dispersive X‐ray spectroscopy (EDS) mapping images (Figure 3b) further confirmed the homogeneous distribution of Fe, C, and O elements over the whole sample. It should be noted that the Fe in Fe_1.0_‐PAC‐24 could be etched by HCl solution (Figure S16 in the Supporting Information), suggesting that Fe might be coordinated with O with weak covalence. Furthermore, X‐ray absorption near‐edge structure (XANES) and EXAFS were conducted to systematically investigate the electronic structures and local coordination environments of the atomically dispersed Fe sites. The Fe K‐edge XANES profiles (Figure 3c) and corresponding valence fitting results (Figure S17a in the Supporting Information) indicated an oxidation state of +2.8 for Fe, consistent with the XPS results (Figure S17b in the Supporting Information). A broad pre‐edge peak around 7114.1 eV was observed and assigned to the 1s→3d transition (inset of Figure 3c), and the weak amplitude of this feature was contributed to the distortion and low symmetry.^[^ ^15^ ^]^ The FT‐EXAFS spectrum for Fe_1.0_‐PA C‐24 exhibited one main peak at ≈1.5 Å, which could be attributed to Fe—O scattering paths (Figure 3d). Importantly, the fingerprinting signal peaks of Fe—Fe interactions (≈2.2 Å) in Fe foil and Fe—O—Fe (≈2.5 Å) in FeO and FeOOH could not be observed in the curve of Fe_1.0_‐PAC‐24.^[^ ^16^ ^]^ The WT contour plots (Figure 3g) demonstrated that only one intensity maximum occurred (4.2 Å^−1^, 1.5 Å) in the Fe_1.0_‐PAC‐24, without Fe—Fe and Fe—O—Fe signals (compared to Fe foil, FeO, and FeOOH), further proving that Fe atoms were atomically dispersed.^[^ ^17^ ^]^ Additionally, the best fitting result of the obtained EXAFS data revealed that the coordination number of Fe was ≈5 (Figure 3e; and Table S3 in the Supporting Information).

To verify the most reasonable coordination structure, six possible Fe‐doped graphene oxide models (M1‐M6 in Figure S18 in the Supporting Information) were considered, in which Fe was coordinated with hydroxyl ligand and epoxy or carbonyl groups in the graphene oxide. As previous studies demonstrated that the oxoiron species usually act as reactive intermediates in the redox reactions, the terminal oxygen connecting to Fe was also included.^[^ ^16^, ^17^ ^]^ The XANES of each model was calculated and then compared with the experimental spectrum of the Fe_1.0_‐PAC‐24 (Figure 3f). It turned out that the theoretically calculated spectrum of M3 (HO—Fe=O, inset of Figure 3f) exhibited similar features to the experimental one, particularly for the shape of the curve and the position of the peak at about 7 115 eV. Thus, the M3 was selected as the model for discussion below.

### 2.3 Characterization of Fe_1.0_‐PAC after H_2_O_2_ Treatment

**Figure 4:**
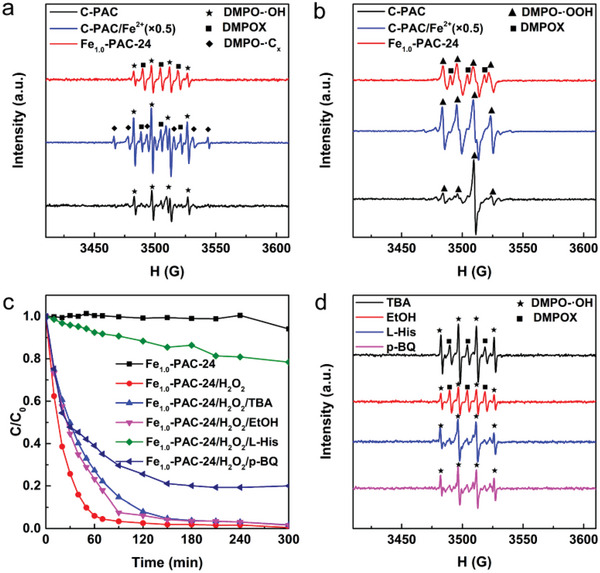
DMPO‐trapped EPR spectra of C‐PAC/H_2_O_2_, C‐PAC/Fe^2+^/H_2_O_2_, and Fe_1.0_‐PAC‐24/H_2_O_2_ systems in a) water and b) ethanol. c) Comparison of RhB degradation rate with or without inhibitors. d) DMPO‐trapped EPR spectra of Fe_1.0_‐PAC‐24/H_2_O_2_ system in water with addition of TBA, EtOH, L‐His, and p‐BQ.

To investigate the underlying activation mechanism toward H_2_O_2_ on Fe_1.0_‐PAC‐24, electron paramagnetic resonance (EPR) experiments were first performed to identify the in situ generated ROS. As shown in **Figure** **4**a,b; and Figure S19 in the Supporting Information, the signal intensities of DMPO–·OH, DMPO–·OOH, and TEMP–^1^O_2_ adducts were similar in both Fe_1.0_‐PAC‐24/H_2_O_2_ and C‐PAC/H_2_O_2_ systems, while the RhB degradation rate in the Fe_1.0_‐PAC‐24/H_2_O_2_ system was far higher than that in the C‐PAC/H_2_O_2_ system, indicating that the three kinds of common ROS (·OH, ·OOH, and ^1^O_2_) were not the major active species in the Fe_1.0_‐PAC‐24/H_2_O_2_ system. In sharp contrast, the signal intensities of the above ROS in the C‐PAC/Fe^2+^/H_2_O_2_ system were much stronger and an extra sextet signal of DMPO‐·C*_x_* adduct appeared (Figure 4a; and Figure S19b in the Supporting Information),^[^ ^18^ ^]^ suggesting that ·OH was the dominating ROS in this system. Interestingly, the specific triplet signals of DMPOX attributed to the breakage of C=N in DMPO could be detected in the Fe_1.0_‐PAC‐24/H_2_O_2_ system (Figure 4a,b),^[^ ^19^ ^]^ implying that a new Fe‐based ROS might be generated. As shown in Figure 4c, the quenching experiment results also demonstrated that ·OH was not the major ROS, which distinguishes this from a traditional Fenton reaction. It was observed that the RhB degradation rate was slightly decreased in the presence of tert‐butanol (TBA) or ethanol (EtOH), while L‐histidine (L‐His) and p‐benzoquinone (p‐BQ) greatly hindered the degradation process. These results indicated that L‐His and p‐BQ could quench the Fe‐based ROS, consistent with the results of DMPO trapped EPR experiments after adding TBA, EtOH, L‐His, and p‐BQ (Figure 4d). The triplet signals of DMPOX disappeared after addition of L‐His or p‐BQ, but were still present after the addition of TBA or EtOH.

**Figure 5:**
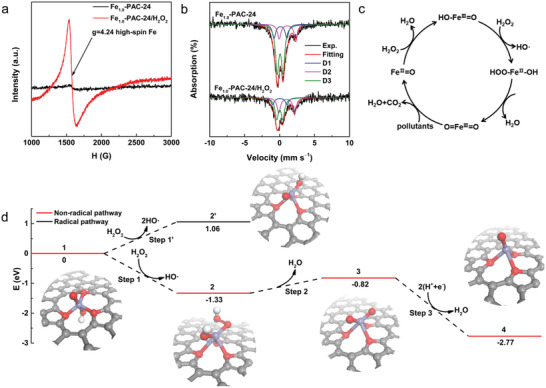
a) EPR spectra of the Fe_1.0_‐PAC‐24 and after adding H_2_O_2_ at 100 K. b) ^57^Fe Mössbauer spectra of the Fe_1.0_‐PAC‐24 and with H_2_O_2_. c) Proposed reaction pathway. d) DFT calculation of nonradical pathway (red line) and radical pathway (black line).

The identification of in situ generated Fe‐based ROS is vital for a deeper understanding of the regeneration process. Quasisitu frozen EPR experiments (**Figure** **5**a; and Figure S20a in the Supporting Information) demonstrated a weak isotropic signal at *g* = 4.24 for fresh Fe_1.0_‐PAC‐24, which were assigned to the high‐spin Fe^III^ species with S = 5/2 (HS‐Fe^III^).^[^ ^20^ ^]^ The intensity sharply increased with the addition of H_2_O_2_, demonstrating that the HS‐Fe^III^ intermediate could be quickly formed and detected in frozen. As previously reported,^[^ ^16^, ^17^ ^]^ we speculate that the oxidative O=Fe=O (HS) moiety is possibly the active intermediate. In addition, the formation of HS‐Fe^III^ could not be inhibited in the presence of common inhibitors (TBA, EtOH, L‐His, and p‐BQ; Figure S20b in the Supporting Information), suggesting the antipoison merit of the active sites.

To further realize the intermediates, in situ zero‐field ^57^Fe Mössbauer spectra were performed to distinguish the change of Fe species in Fe_1.0_‐PAC‐24 during H_2_O_2_ activation. The quadrupole splitting (QS) and isomer shift (IS) parameters are given in Table S4 in the Supporting Information. Figure 5b shows that the ^57^Fe Mössbauer spectra of Fe_1.0_‐PAC‐24 and Fe_1.0_‐PAC‐24 with H_2_O_2_ could be well‐fitted with three doublets (D1, D2, and D3). D1, D2, and D3 represent the intermediate‐spin (IS) Fe species,^[^ ^21^ ^]^ high‐spin (HS) Fe^II^ species,^[^ ^22^ ^]^ and the low‐spin (LS) Fe^III^ species,^[^ ^23^ ^]^ respectively. It was found that the relative area of D2 increased significantly and the relative area of D3 decreased accordingly during the whole reaction, showing that part of LS‐Fe^III^ transformed to HS‐Fe^II^ and reached a balance between Fe^II^ and Fe^III^. It should be noted that the rapid interconversion of Fe^II^/Fe^III^ is beneficial for continuous H_2_O_2_ activation.^[^ ^24^ ^]^ The relative area of D1 remained almost unchanged, revealing that the intermediate‐spin Fe species could be rapidly formed and consumed. According to the above results, a nonradical reaction pathway is therefore supposed in Figure 5c, involving the following steps: 1) HO—Fe^III^ = O (LS) is the initial state, and H_2_O_2_ reacts with HO—Fe^III^=O to form HOO—Fe—OH (IS); 2) HOO—Fe—OH converts to O=Fe^III^=O; 3) O=Fe^III^ =O is reduced to Fe^II^=O (HS) by organic pollutants; 4) final‐state Fe^II^=O is oxidized to HO—Fe^III^=O.

To provide an in‐depth understanding of the mechanism of H_2_O_2_ activation, M3 was selected as a model and DFT calculations were performed. The reaction pathway in Figure 5d was supposed, in which the terminal oxygen reacts with H_2_O_2_ to generate hydroxyl and hydroperoxyl groups first. After the structure relaxation, the hydroxyl group on the other side of the carbon layer departs from the Fe center to form a hydroxyl radical with an energy release of 1.33 eV, implying that the formation of hydroxyl radicals is feasible, consistent with EPR results. The direct hemolysis of the O—O bond at the Fe=O site is also simulated, which requires +1.06 eV to generate O=Fe—OH and hydroxyl radicals (i.e., a radical pathway). Thus, the formation of HOO—Fe—OH intermediate (i.e., a nonradical pathway) is energetically favorable from a simulation perspective. Subsequently, a water molecule may further depart with an energy change of +0.51 eV, forming a highly active species with two terminal oxygen groups. To evaluate its redox reactivity, a hypothetical electrochemical reaction was designed in which the intermediate reacts with two proton–electron pairs. According to the computational hydrogen electrode model, Nørskov et al. supposed that the energy of one proton–electron pair equals the energy of half a hydrogen molecule plus 0.24 eV.^[^ ^25^ ^]^ Herein the energy change to form the final oxo‐Fe species is as negative as −1.95 eV. Considering the enthalpy of vaporization of water (0.42 eV), the free energy change must be lower than −2.0 eV, corresponding to a standard electrode potential higher than 1.0 V. Thus, the intermediate 3 should be a quite strong oxidizing species.

## 3 Conclusion

Conclusively, a strategy of anchoring isolate Fe atoms on commercial PAC to realize cost‐effective PAC regeneration by H_2_O_2_ is presented. In experiments, a 10‐cycle regeneration performance of Fe_1.0_‐PAC with RhB as a representative pollutant was observed. The high regeneration efficiency (70.5–92.7%), low loss rate of absorbent (≈8.25% per cycle), and low H_2_O_2_ dosage (2.31 g g_(PAC)_^−1^) endows the presented Fe_1.0_‐PAC with a bright future in large‐scale wastewater treatment. After detailed characterizations, this outstanding regeneration performance was attributed to the formation of oxidative O=Fe=O intermediates to degrade organic pollutants and fast transformation between Fe^3+^ (LS) and Fe^2+^ (HS).

## 4 Experimental Section

### Materials

All chemicals were obtained from commercial suppliers at analytical grade and used without further purification. Fe(NO_3_)_3_∙9H_2_O, urea, ethanol (EtOH), powdered active carbon (PAC), and 30% H_2_O_2_ solution were obtained from Sinopharm Chemical Reagent Co., Ltd. Rhodamine B, tertiary butanol (TEA), L‐histidine (L‐His), p‐benzoquinone (p‐BQ), 5,5‐dimethyl‐1‐pyrroline N‐oxide (DMPO), and 2,2,6,6‐tetramethylpiperidine (TEMP) were obtained from Shanghai Aladdin Bio‐Chem Technology Co., Ltd. Methylene blue (MB) and crystal violet (CV) were obtained from Adamas‐Beta (Shanghai) Chemical Reagents Co., Ltd.

### Fe*_x_*‐PAC Preparation

For the synthesis of Fe‐PAC with 1.0% Fe (Fe_1.0_‐PAC), a stock solution containing 0.01 mmol L^−1^ Fe(NO_3_)_3_ and 0.06 mmol L^−1^ urea was first prepared by dissolving Fe(NO_3_)_3_·9H_2_O and urea into ethanol. An activated carbon suspension was prepared by mixing 2 g of PAC with 200 mL of ethanol and sonicated for 30 min until good dispersion was achieved. Then 40 mL of precursor solution was added dropwise into the PAC suspension under vigorous stirring overnight and then centrifuged to collect the precursor (Fe(urea)_6_‐PAC). The as‐prepared Fe(urea)_6_‐PAC was heated up at 10 °C min^−1^ in a tube furnace to 800 °C under a gas flow of 60 standard cubic centimeters per minute (sccm) N_2_ and maintained for 1 h, to obtain the final products. For Fe‐PAC with 0.67% (Fe_0.67_‐PAC), the concentrations of Fe(NO_3_)_3_ and urea were adjusted to 0.005 and 0.03 mmol L^−1^, respectively.

Due to the limited absorption capacity of PAC for Fe(urea)_6_, the precursor with high Fe loading was obtained by an immersion process. For the synthesis of Fe‐PAC with 4.8% Fe (Fe_4.8_‐PAC), a stock solution containing 0.005 mmol L^−1^ Fe(NO_3_)_3_ and 0.03 mmol L^−1^ urea was first prepared by dissolving Fe(NO_3_)_3_·9H_2_O and urea into 80 mL of ethanol. Then, 1 g of PAC was added into the solution and the suspension was dried under vigorous stirring. The PAC with Fe(urea)_6_ was obtained and heated up in a tube furnace to 800 °C under a gas flow of 60 standard cubic centimeters per minute (sccm) N_2_ and maintained for 1 h, to obtain the final products. For Fe‐PAC with 9.9% Fe (Fe_9.9_‐PAC), the amount of PAC was adjusted to 0.5 g. The mass fraction of Fe in Fe‐PAC was determined by Inductively Coupled Plasma Optical Emission Spectrometer (ICP‐OES).

### Fe_1.0_‐PAC‐y Preparation

For the synthesis of Fe_1.0_‐PAC with different activation times, 100 mg of Fe_1.0_‐PAC were added into 100 mL of 0.6 mol L^−1^ H_2_O_2_ solution and stirred for a certain time (*y* = 2, 4, 8, 12, and 24 h). The obtained samples were denoted as Fe_1.0_‐PAC‐y. The mass fraction of Fe in Fe_1.0_‐PAC after activation was determined by ICP‐OES.

### Characterization

HRTEM images and EDS mapping were obtained using a JEOL JEM‐2100F field emission electron microscope operated at 200 kV. AC‐HAADF‐STEM images were taken with a Themis Z scanning/transmission electron microscope operated at 300 kV, equipped with a probe spherical aberration corrector. XRD was carried out on a Rigaku D/max 2500Pc X‐ray powder diffractometer with monochromatized Cu K*α* radiation (*λ* = 1.5418 Å) at scan rate of 10° min^−1^. The XPS spectra were obtained on a Thermo Scientific Escalab 250Xi with an Al K*α* source (1486.8 eV). ICP‐OES was taken on an Agilent ICP 720. The BET surface areas were measured using a Micromeritics ASAP2020 at 77 K. EPR measurements were carried out on a Bruker E500 EPR spectrometer at 100 and 298 K.^57^Fe Mössbauer spectroscopy was recorded on a Topologic Systems MFD‐500AV spectrometer with alternating constant acceleration of the *γ*‐source. Fe K‐edge X‐ray absorption spectroscopy (XAS) were conducted at beamline 14W1 of the Shanghai Synchrotron Radiation Facility (SSRF), China, and the XAFCA beamline of Singapore Synchrotron Light Source. Data were recorded in fluorescence mode for Fe K‐edge.

### XAFS Analysis

All XAFS data were analyzed using the program Demeter.^[^ ^26^ ^]^ For all samples, the EXAFS oscillations were extracted from the normalized XAS spectra by subtracting the atomic background using a cubic spline fit to *k* ^3^‐weighted data, where *k* is the photoelectron wave number. The *χ*(*k*) functions were then Fourier Transformed into R‐space. The Fourier‐Transform window was in the *k* range 2–10 Å for Fe. Simulations of the XANES were performed using the finite difference method, as implemented within the Finite Difference Method Near Edge Scattering (FDMNES) package, using a free form SCF potential of radius 6.0 Å around the absorbing atom. Broadening contributions due to the finite mean‐free path of the photoelectron and to the core‐hole lifetime were accounted for using an arctangent convolution.

### Quantification of Pollutants

The RhB, MB, and CV concentrations were detected with a PERSEE TU‐1810 UV–vis spectrometer. To obtain the calibration curves, RhB of known concentration was measured using UV–vis spectroscopy at *λ* = 554 nm. The detector wavelength was set at *λ* = 665 nm for MB, and at *λ* = 583 nm for CV. The calibration curves of RhB, MB, and CV are shown in Figure S6 in the Supporting Information. Based on the linear relationship between the signal intensity and concentration, RhB, MB, or CV concentrations of the samples could be obtained.

### Adsorption Isotherm

To obtain adsorption isotherms, 50 mL of RhB solutions at different concentrations (*C*_0_ = 10, 20, 30, 40, 50, 60, 80, and 100 ppm) were mixed with 10 mg of sample (C‐PAC or Fe_1.0_‐PAC). The adsorption process was carried out in a shaker at 25 °C and 240 rpm for 24 h. The concentration in solution at equilibrium (*C*_e_) was measured with a PERSEE TU‐1810UV–vis spectrometer at *λ* = 554 nm and the concentration of solids at equilibrium (*Q*_e_) was calculated using Equation (1)

(1) $$Q_{e}=\frac{\left(C_{0}-C_{e}\right)\times \;0.05}{0.01}$$

Then, the adsorption isotherms were analyzed by Langmuir Equation (Equation (2)), where *Q*_max_ (mg g^−1^) is the maximum amount of RhB adsorbed within a monolayer. *K* (L mg^−1^) is the Langmuir dissociation constant related to the adsorption energy

(2) $$Q_{e}=\frac{Q_{\max}\times K\times C_{e}}{1+K\times C_{e}}$$

The adsorption isotherms of MB and CV were drawn using the same methods (shown in Figures S10 and S11 in the Supporting Information).

### Adsorption Kinetics

To evaluate the adsorption kinetics, 50 mL of 40 ppm RhB solution was mixed with 10 mg of sample (C‐PAC or Fe_1.0_‐PAC). The mixture was shaken at 25 °C and 240 rpm, and at time intervals (*t* = 5, 10, 15, 20, 25, 30, 40, 50, and 60 min), liquid samples were taken in order to analyze the variation of the RhB concentration.

### Catalytic Performance Evaluation

To evaluate the catalytic kinetics, RhB degradation experiments were carried out. For Fe_1.0_‐PAC or Fe_1.0_‐PAC‐y, 10 mg of sample were added into 50 mL of 80 ppm RhB solution without pH adjustment and the suspension was shaken at 25 °C and 240 rpm for 24 h to approach adsorption–desorption equilibrium. Then, 3 mL of 30% H_2_O_2_ solution (H_2_O for blank experiments) was added into the suspension to trigger the catalytic reaction. A certain volume of sample was withdrawn at time intervals and immediately quenched with saturated Na_2_SO_3_ solution. The remaining concentration of RhB was measured with a PERSEE TU‐1810 UV–vis spectrometer at *λ* = 554 nm. For C‐PAC or C‐PAC with different concentrations of Fe^2+^ (pH was adjusted to ≈3), the initial concentration of RhB solution was 100 ppm.

The mass‐based degradation rate (*v*, mg g^−1^ min^−1^) was calculated with Equation (3), where *C*_0_ and *C*_t_ were the initial concentration and concentration at a certain time *t* (mg L^−1^), *m*_cat._ was the mass of catalyst (g), and *t* was the reaction time (min)

(3) $$v=\frac{\left(C_{0}-C_{t}\right)\times \;0.05}{m_{\mathrm{cat}.}\times t}$$

The TOF_Fe_ (h^−1^) was calculated using Equation (4), where *m*_Fe_ was the mass of Fe loading (mg) and *t* was the reaction time (h)

(4) $${\mathrm{TOF}}_{\mathrm{Fe}}=\frac{\left(C_{0}-C_{t}\right)\times \;0.05}{m_{\mathrm{Fe}}\times t}$$

The pseudofirst‐order kinetic *k* (min^−1^) was calculated with Equation (5)

(5) $$\ln\frac{C_{t}}{C_{0}}=-kt$$

### Regeneration Performance Evaluation

The adsorption/regeneration process was conducted as shown in Figure 1. According to industrial regeneration requirements, the adsorption amount was set at ≈30–50% of the saturation adsorption. In a typical vial experiment, 10 mg of sample was dispersed in 50 mL of 20 ppm RhB aqueous solution without pH adjustment. The adsorption process was carried out in a shaker at 25 °C and 240 rpm for 30 min. The suspension was collected and centrifuged at 12 000 rpm for 8 min, then the supernatant was sampled and analyzed. For the regeneration experiment, the RhB adsorbed sample was separated and stirred in 100 mL of 0.6 mol L^−1^ H_2_O_2_ solution for 24 h. For the C‐PAC/Fe^2+^/H_2_O_2_ system, an additional 1.5 mg of Fe^2+^ was needed. After regeneration, the sample was collected by filtration, washed with deionized water and dried at 60 °C for the next cycle. The dosage of H_2_O_2_ was measured with a UV–vis spectrometer and the mass loss of PAC was determined by weighing. For MB and CV, the concentrations were set at 20 and 30 ppm, respectively.

### Quantification of H_2_O_2_ Concentration

The H_2_O_2_ concentration was determined by a traditional Ti(SO_4_)_2_ titration method based on the principle that a yellow solution of H_2_TiO_4_ will be produced (Equation (6)). Thus, the concentration of H_2_TiO_4_ can be measured by UV–vis spectroscopy at *λ* = 408 nm

(6) $${\mathrm{Ti}}^{4+}+H_{2}O_{2}+2H_{2}O=H_{2}{\mathrm{TiO}}_{4}+4H^{+}$$

Ti(SO_4_)_2_ solution (2 mmol L^−1^) was prepared by dissolving 0.2 mmol Ti(SO_4_)_2_ in 100 mL of 1 mol L^−1^ H_2_SO_4_ solution. To obtain the calibration curve, 2 mL of H_2_O_2_ of known concentration was added to 2 mL of Ti(SO_4_)_2_ solution and measured using UV–vis spectroscopy (the calibration curve is shown in Figure S8 in the Supporting Information). Based on the linear relationship between the signal intensity and H_2_TiO_4_ concentration, the H_2_O_2_ concentration of the samples could be obtained.

### H_2_O_2_ Dosage

The dosage of H_2_O_2_ was calculated using Equation (7), where the *C*_initial_ and *C*_final_ were the initial and final H_2_O_2_ concentration (mg L^−1^), and *V* was the volume of regeneration solution (L)

(7) $$H_{2}O_{2\;}\left(\mathrm{mg}\right)=\left(C_{\mathrm{initial}}-C_{\mathrm{final}}\right)\times V$$

### Quenching Experiments

In a typical experiment, 10 mg of Fe_1.0_‐PAC‐24 were added into 50 mL of 80 ppm RhB solution without pH adjustment and the suspension was shaken at 25 °C and 240 rpm for 24 h to approach adsorption–desorption equilibrium. The ROS scavengers (TEA and EtOH for ·OH, L‐His for ^1^O_2_, and p‐BQ for ·OOH) were dissolved and the concentrations of TEA, EtOH, L‐His, or p‐BQ were 1, 1, 0.25, and 0.04 mol L^−1^, respectively. Then, 3 mL of 30% H_2_O_2_ solution was added into the suspension to trigger the catalytic reaction. A certain volume of sample was withdrawn at a certain time interval and immediately quenched with saturated Na_2_SO_3_ solution. The remaining concentration of RhB was measured with a PERSEE TU‐1810 UV–vis spectrometer at *λ* = 554 nm.

### DMPO‐Trapped EPR Signals

Typically, 10 mg of Fe_1.0_‐PAC‐24, C‐PAC, or C‐PAC with Fe^2+^ and 0.6 mL of 30% H_2_O_2_ were added to 10 mL of deionized water (for detecting ·OH) or ethanol (for detecting ·OOH). After a period of reaction time, 100 µL of the above suspension and 20 µL of DMPO were mixed for 5 min. Then, the solution was sucked into a capillary to carry out EPR at 25 °C.

For quenching experiments, the only difference was that when mixing the samples and H_2_O_2_ solution, ROS scavengers were also added. The concentrations of TEA, EtOH, L‐His, or p‐BQ were 1, 1, 0.25, and 0.04 mol L^−1^, respectively.

### TEMP‐Trapped EPR Signals

Typically, 10 mg of Fe_1.0_‐PAC‐24, C‐PAC, or C‐PAC with Fe^2+^ and 0.6 mL of 30% H_2_O_2_ were added to 10 mL of deionized water. After a period of reaction, 100 µL of the above suspension and 20 µL of 0.1 mol L^−1^ TEMP solution were mixed for 5 min. Then, the solution was sucked into a capillary to carry out EPR at 25 °C.

### High‐Spin Fe EPR Signals

Typically, 10 mg of Fe_1.0_‐PAC‐24 with/without 50 µL of 30% H_2_O_2_ were added into a quartz tube and held for 5 min. The tube was then frozen by liquid nitrogen and the EPR was carried out at 100 K. The EPR signal of the quartz tube was also tested without Fe_1.0_‐PAC‐24.

For quenching experiments, 50 µL of 30% H_2_O_2_ containing 1, 1, 0.25, or 0.04 mol L^−1^ of TEA, EtOH, L‐His or p‐BQ were mixed with the Fe_1.0_‐PAC‐24.

### Calculation Details

All the spin‐polarized density functional theory calculations were performed by employing the Vienna ab initio simulation package (VASP).^[^ ^27^ ^]^ Perdew–Burke–Ernzerhof (PBE)^[^ ^28^ ^]^ functional of generalized gradient approximation (GGA) with projector augmented wave (PAW)^[^ ^29^ ^]^ was used to describe the electronic structures. The energy cutoff of the plane wave was set as 450 eV. The convergence criteria for geometry optimization was 0.03 eV Å^−1^ in force. The Brillouin zone sampling only considered the Gamma point. Simulation models were based on a 8 × 4√3 graphene supercell, with a size of 19.68 × 17.04 Å in the *x* and *y* direction. A vacuum layer of 20 Å in the *z* direction was added to avoid the interaction between neighboring images in periodic boundary condition. Several carbon atoms were removed to introduce oxygen containing functional groups. Iron atoms coordinated with oxygen groups. Hubbard U was applied for Fe, with U value of 4.0 eV.

## Conflict of Interest

The authors declare no conflict of interest.

## Supporting information

Supporting Information

## Data Availability

The data that support the findings of this study are available from the corresponding author upon reasonable request.
