# Supplementary material for: Cost‐Effective H2O2‐Regeneration of Powdered Activated Carbon by Isolated Fe Sites
Source: Adv Sci (Weinh). 2022 Nov 18;10(2):2204079. doi: 10.1002/advs.202204079 (PMC9839841; doi:10.1002/advs.202204079)
Supplement: Supplementary file 1 — Supporting Information [file ADVS-10-2204079-s001.pdf]

ADVANCED SCIENCE
Open Access

Supporting Information

for *Adv. Sci.*, DOI 10.1002/advs.202204079

Cost-Effective H_2O_2-Regeneration of Powdered Activated Carbon by Isolated Fe Sites

*Xu Chen, Ziqi Tian, Qihao Yang, Linjuan Zhang, Qiu Yang, Liang Chen and Zhiyi Lu**

DOI: 10.1002/advs.202204079

Supporting Information

Cost-Effective H_2O_2-Regeneration of Powdered Activated Carbon by Isolated Fe Sites

*Xu Chen, Ziqi Tian, Qihao Yang, Linjuan Zhang, Qiu Yang, Liang Chen, and Zhiyi Lu**

Key Laboratory of Advanced Fuel Cells and Electrolyzers Technology of Zhejiang Province, Qianwan Institute of CNITECH, Ningbo Institute of Materials Technology and Engineering, Chinese Academy of Sciences
Ningbo, Zhejiang 315201, P. R. China
E-mail: luzhiyi@nimte.ac.cn

Dr. X. Chen, Prof. Z. Tian, Dr. Q. Yang, Prof. L. Chen, Prof. Z. Lu
University of Chinese Academy of Sciences
Beijing 100049, P. R. China

Prof. L. Zhang
Key Laboratory of Interfacial Physics and Technology, Shanghai Institute of Applied Physics, Chinese Academy of Sciences
Shanghai 201800, P. R. China

Dr. Q. Yang
Ningbo New Material Testing and Evaluation Center Co., Ltd, Ningbo New Materials Innovation Center
Ningbo, Zhejiang 315201, P. R. China

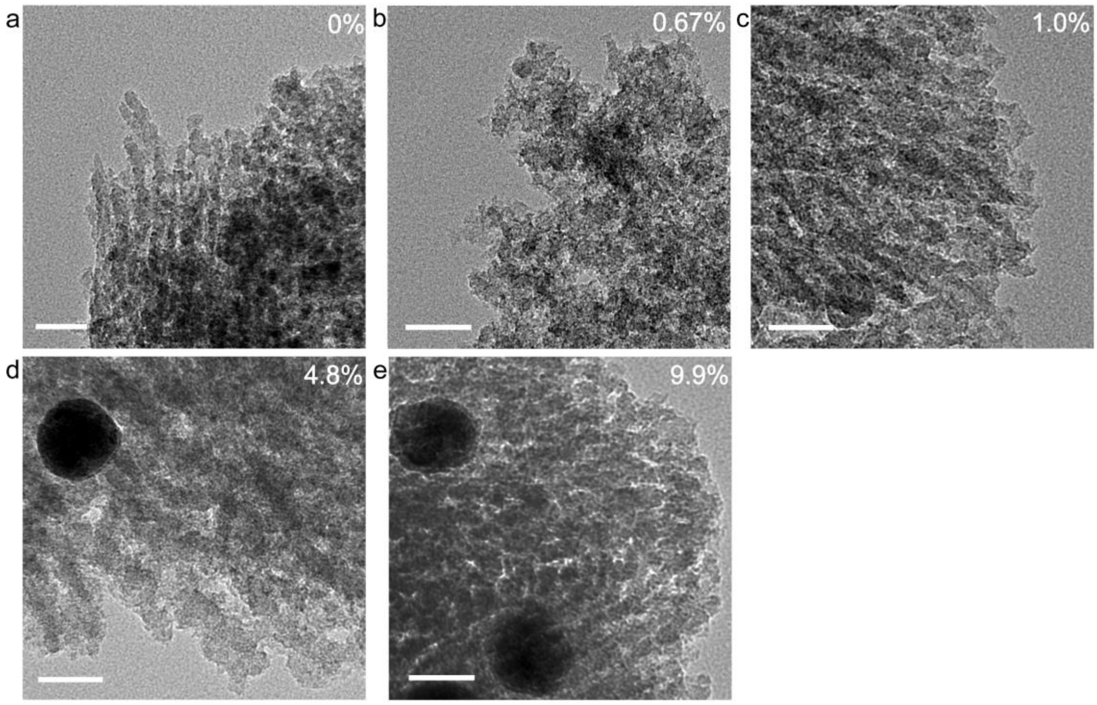

Figure S1. SEM images of the samples with different Fe contents (a) 0%, (b) 0.67%, (c) 1.0%, (d) 4.8% and (e) 9.9%. (scale bar 50 nm)

Note: The Fe-PAC with Fe content ≤1 wt% exhibited no observable nanoparticles, while further increasing the Fe content to 4.8%, the Fe-based nanoparticles with a diameter of ~50 nm was formed. And the number of nanoparticles increased with increasing Fe content.

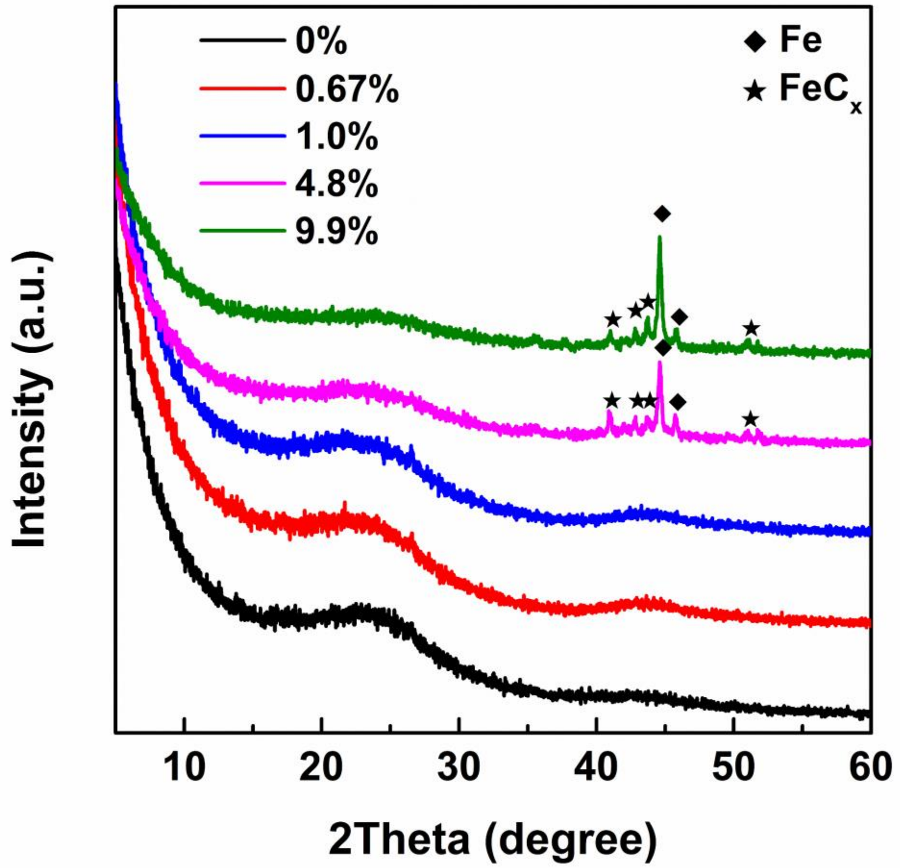

Figure S2. XRD patterns of the samples with different Fe contents from 0% to 9.9%.

Note: For the Fe-PAC with Fe content ≤1 wt%, only a broad peak at 2θ=25° ((002) plane of carbon) could be observed from their X-ray diffraction (XRD) patterns. However, when the Fe content was increased to 4.8 wt%, some sharp peaks belonging to Fe or FeC_x would appear.

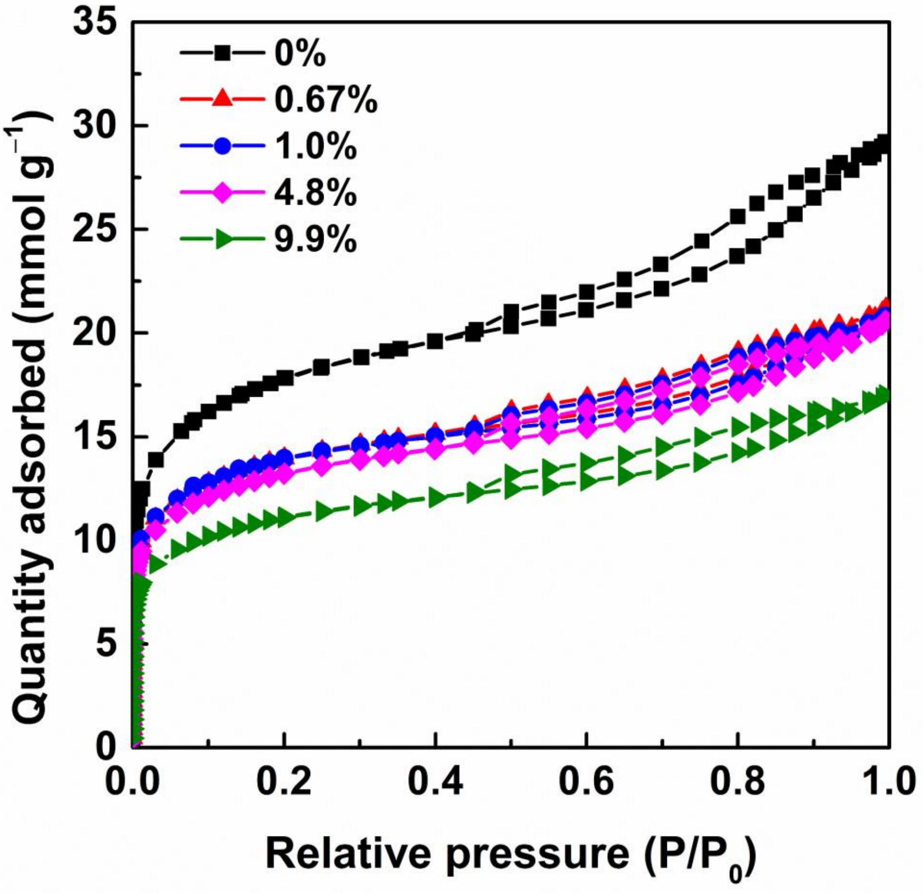

Figure S3. N_2 adsorption-desorption isotherms at 77 K of the samples with different Fe contents from 0% to 9.9%.

Note: The specific surface area of the samples with different Fe contents from 0% to 9.9% were measured by the multipoint Brunauer-Emmett-Teller (BET) procedure from N_2 adsorption-desorption isotherms at 77 K. The specific surface areas decreased with increase of Fe content and were 1389.6, 1119.6, 1121.7, 1061.2 and 894.1 $m^2\ g^{-1}$.

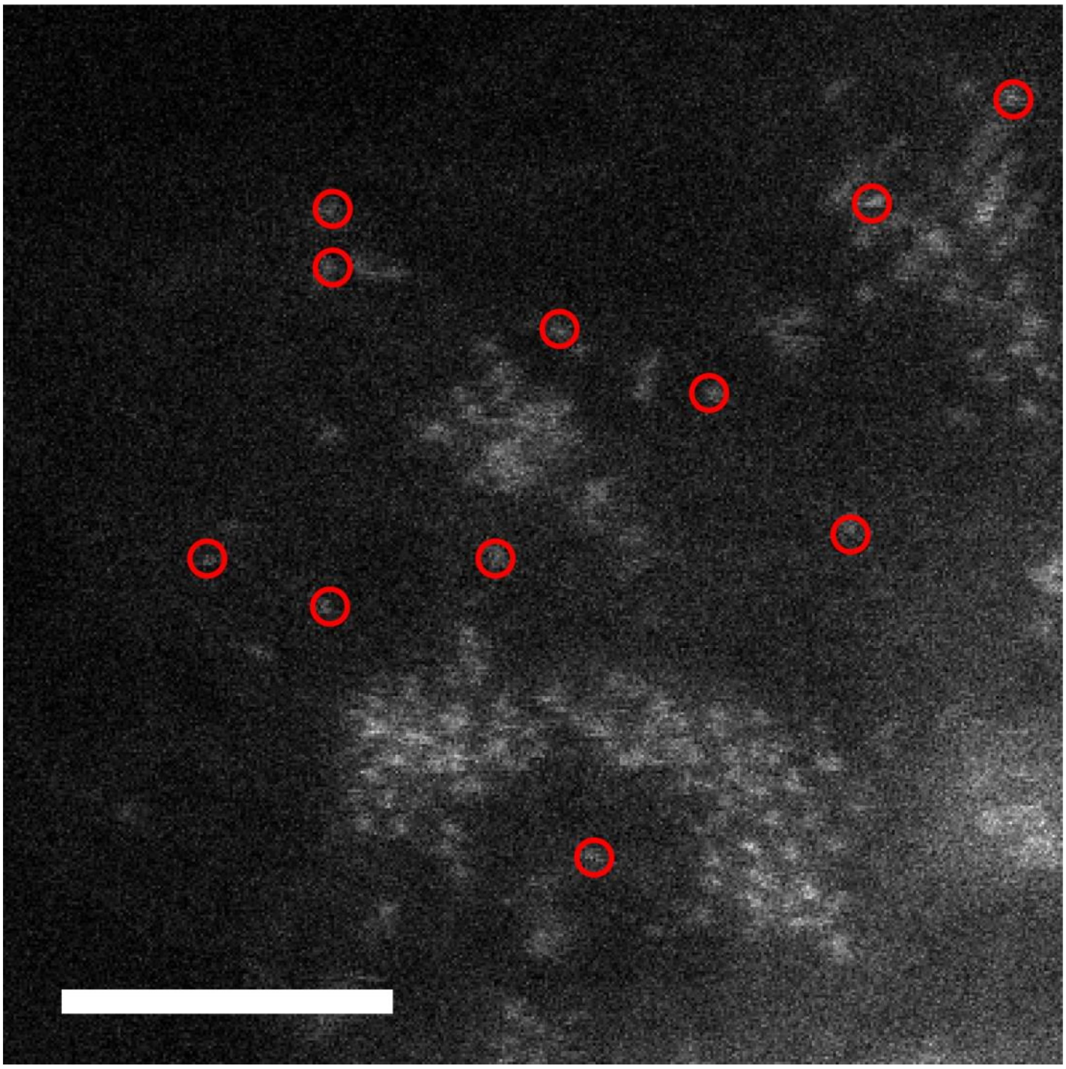

Figure S4. AC-HAADF-STEM image of $Fe_{1.0}$-PAC. (scale bar 2 nm)

Note: Fe single atoms and clusters coexisted in the $Fe_{1.0}$-PAC.

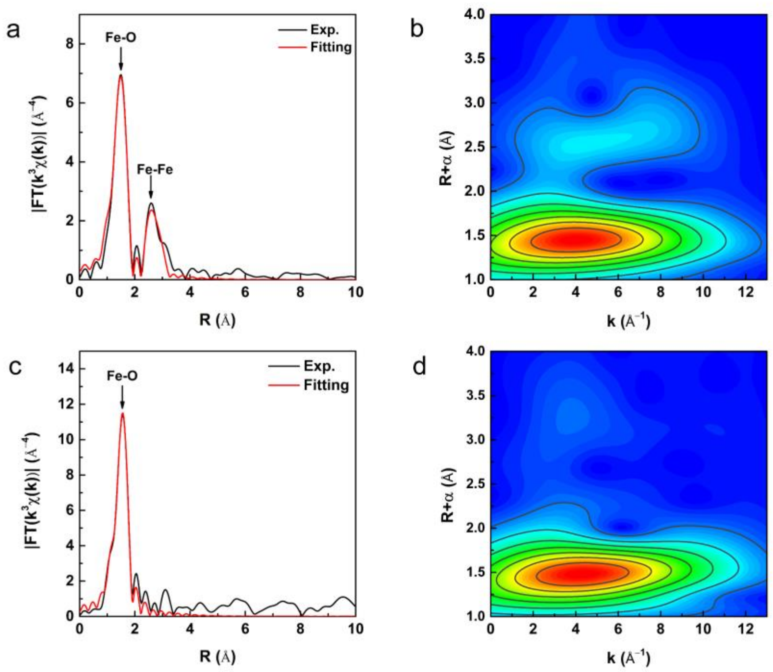

Figure S5. (a) FT k^3-weighted χ(k)-function of the EXAFS spectra and (b) WT of the Fe K-edge for $Fe_{1.0}$-PAC before regeneration. (c) FT k^3-weighted χ(k)-function of the EXAFS spectra and (d) WT of the Fe K-edge for $Fe_{1.0}$-PAC before regeneration.

Note: The Fourier transform-EXAFS (FT-EXAFS) spectra for $Fe_{1.0}$-PAC before regeneration exhibited two main peaks at ~1.5 Å and ~2.5 Å, which could be attributed to Fe-O and Fe-Fe scattering paths. The wavelet transform (WT) contour plots demonstrate that two intensity maximum occurs at about (4.2 $Å^{-1}$, 1.5 Å) and (4.8 $Å^{-1}$, 2.5 Å). The FT-EXAFS spectra for $Fe_{1.0}$-PAC after regeneration exhibited one main peak at ~1.5 Å, which could be attributed to Fe-O scattering paths. The WT contour plots demonstrate that one intensity maximum occurs at about (4.2 $Å^{-1}$, 1.5 Å).

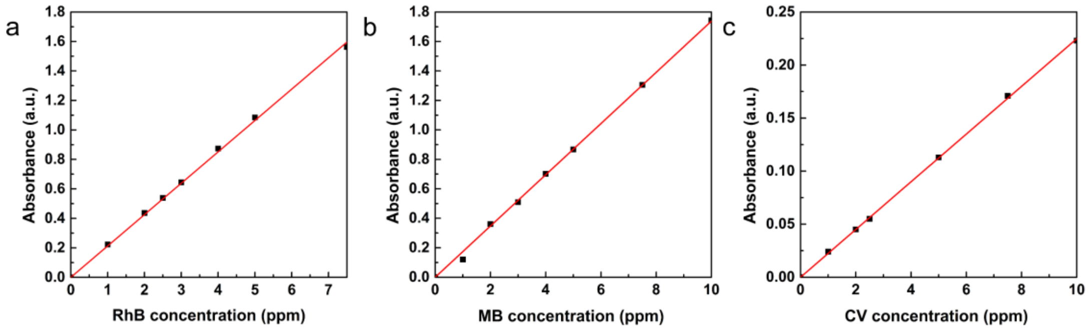

Figure S6. Calibration curve of RhB (a), MB (b) and CV (c) solution.

Note: The calibration curve of RhB solution was measured at λ=554 nm by an UV-vis spectroscopy. Absorbance is linearly related to RhB concentration in the range of 0 to 7.5 ppm. The slope value is 0.213 and adj. R-square is 0.9995.

$$Abs.(a.u.) = 0.213 \times C_{RhB}(ppm)$$

The calibration curve of MB solution was measured at λ=665 nm by an UV-vis spectroscopy. Absorbance is linearly related to MB concentration in the range of 0 to 10 ppm. The slope value is 0.174 and adj. R-square is 0.9994.

$$Abs.(a.u.) = 0.174 \times C_{MB}(ppm)$$

The calibration curve of CV solution was measured at λ=583 nm by an UV-vis spectroscopy. Absorbance is linearly related to CV concentration in the range of 0 to 10 ppm. The slope value is 0.0225 and adj. R-square is 0.9998.

$$Abs.(a.u.) = 0.0225 \times C_{CV}(ppm)$$

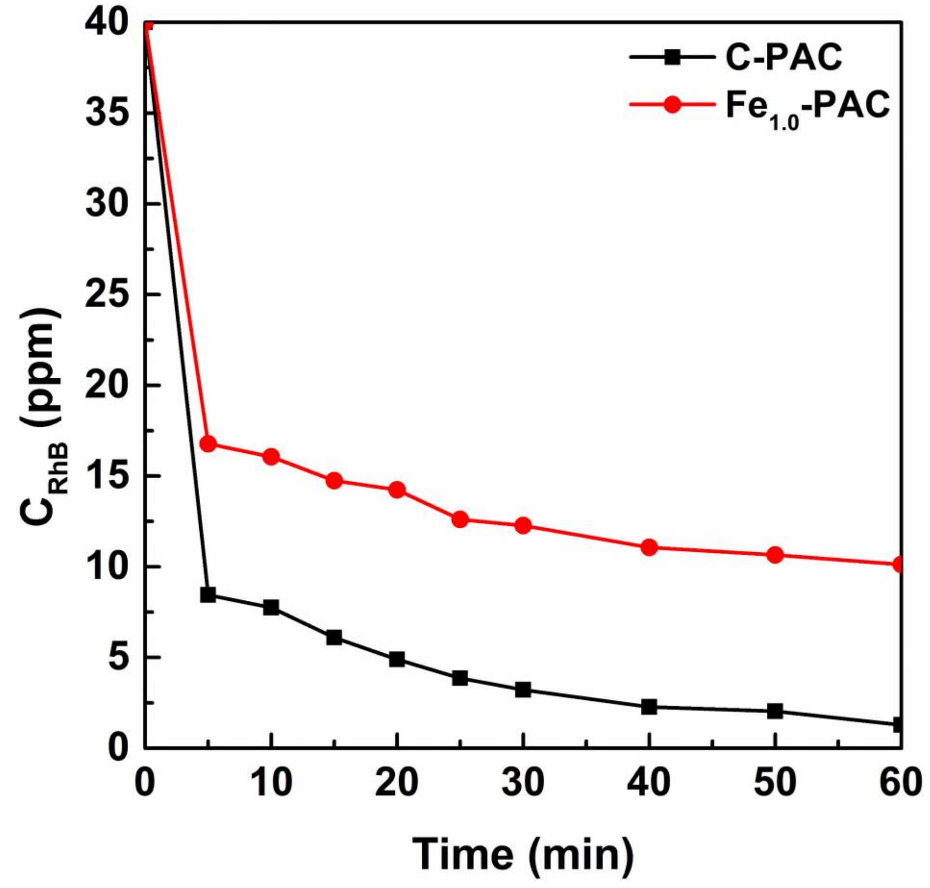

Figure S7. Adsorption kinetics of RhB on C-PAC and $Fe_{1.0}$-PAC.

Note: The C-PAC and $Fe_{1.0}$-PAC had similar adsorption behavior from adsorption curve. The RhB was rapidly adsorbed in the first 5 min and then the adsorption rate slowed down. This phenomenon indicated that the introduction of Fe hardly changed the surface structure of PAC. The adsorption kinetics of RhB on $Fe_{1.0}$-PAC is slower than C-PAC, which was due to the reduce of the surface area.

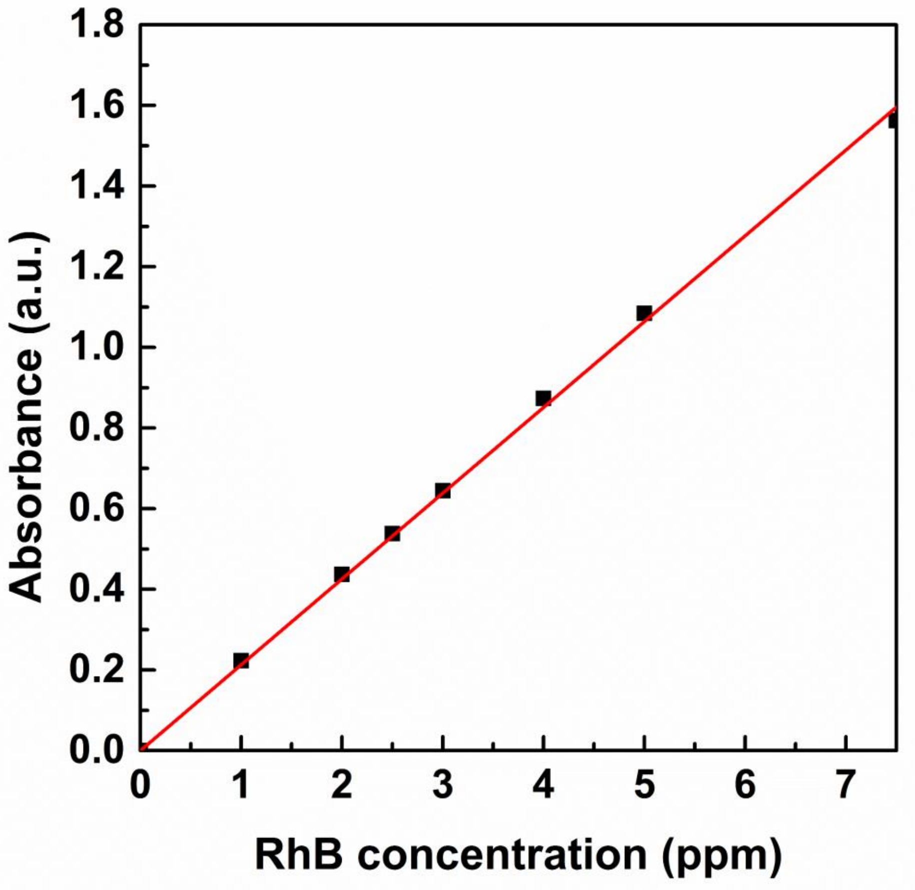

Figure S8. Calibration curve of H_2O_2 solution in neutral media.

Note: The calibration curve of H_2O_2 solution with $Ti(SO_4)_2$ solution was measured at λ=408 nm by an UV-vis spectroscopy. Absorbance is linearly related to H_2O_2 concentration in the range of 0 to 34 ppm. The slope value is 0.0104 and adj. R-square is 0.9995.

$$Abs.(a.u.) = 0.0104 \times C_{H_2O_2}(ppm)$$

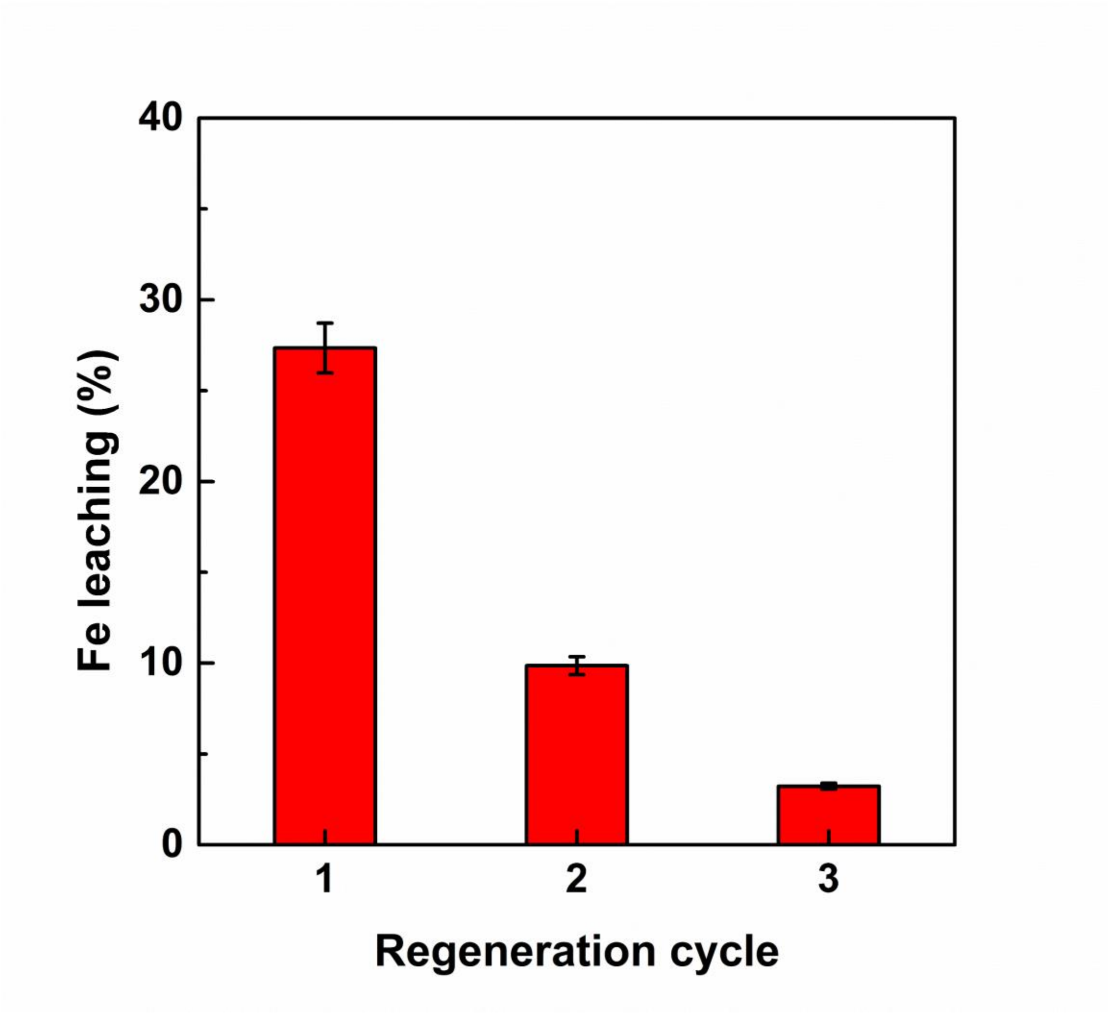

Figure S9. Fe leaching rates during regeneration.

Note: The iron in the $Fe_{1.0}$-PAC would leach out during the Fenton reaction, due to dissolution of the Fe sites and destruction of $Fe_{1.0}$-PAC. The average Fe leaching was 13.5% in 3 regeneration cycles (corresponding to 4 adsorption cycles) and the leaching rate would decrease sharply with the increase of regeneration cycles.

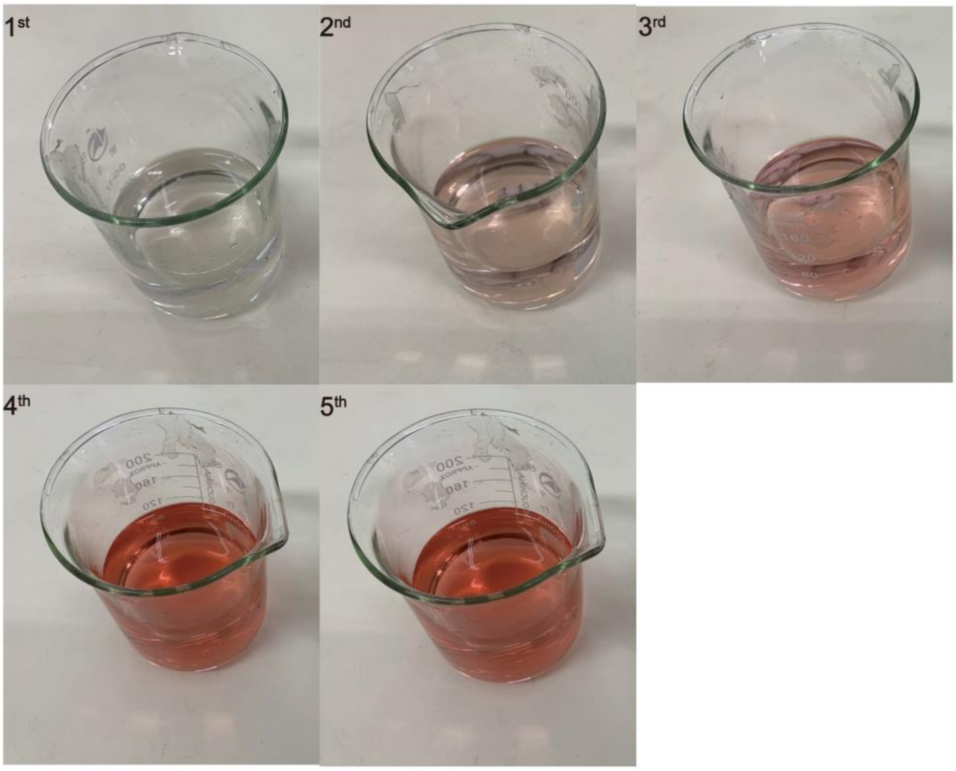

Figure S10. The solution after C-PAC regeneration.

Note: The RhB desorbed from the C-PAC during the regeneration process.

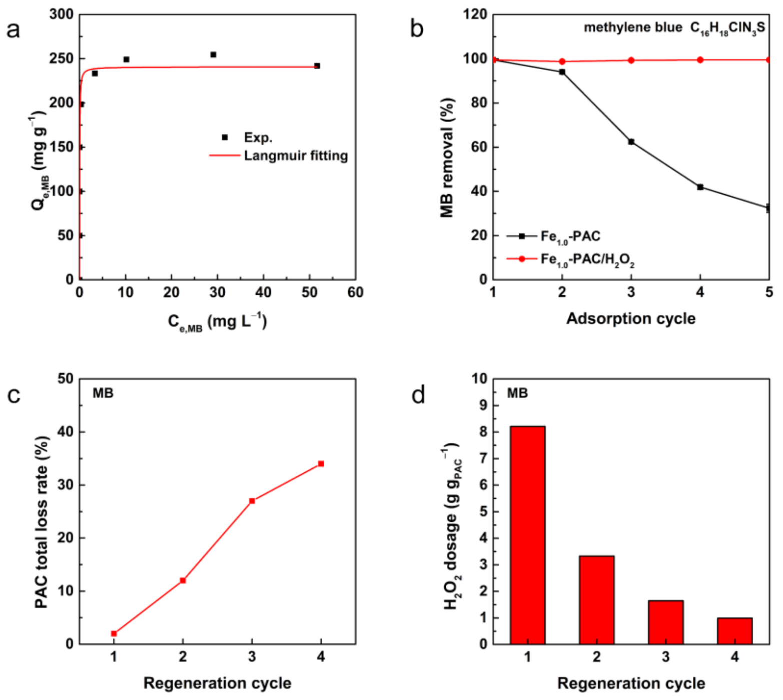

Figure S11. (a) Adsorption isotherm for MB on $Fe_{1.0}$-PAC. (b) Comparison of cyclic MB removal performance in $Fe_{1.0}$-PAC and $Fe_{1.0}$-PAC with H_2O_2 regeneration systems. [MB]=20 ppm (~40% of saturation adsorption), adsorption time=30 min. (c) PAC loss and (d) H_2O_2 dosage during regeneration process for $Fe_{1.0}$-PAC adsorbing MB system.

Note: It is observed that the maximum MB adsorption capacity of $Fe_{1.0}$-PAC (qm) was ~240 mg g^{-1} by the Langmuir isotherm models. The removal efficiency of $Fe_{1.0}$-PAC could be maintained at 98.8% with H_2O_2-regenerationin every cycle, while that of $Fe_{1.0}$-PAC without H_2O_2-regeneration decreased gradually down to 32.4%. The PAC loss rate of $Fe_{1.0}$-PAC was 34.0% and average H_2O_2 consumption was 3.54 g/$g_{(PAC)}$ after 4 regeneration cycles (corresponding to 5 adsorption cycles).

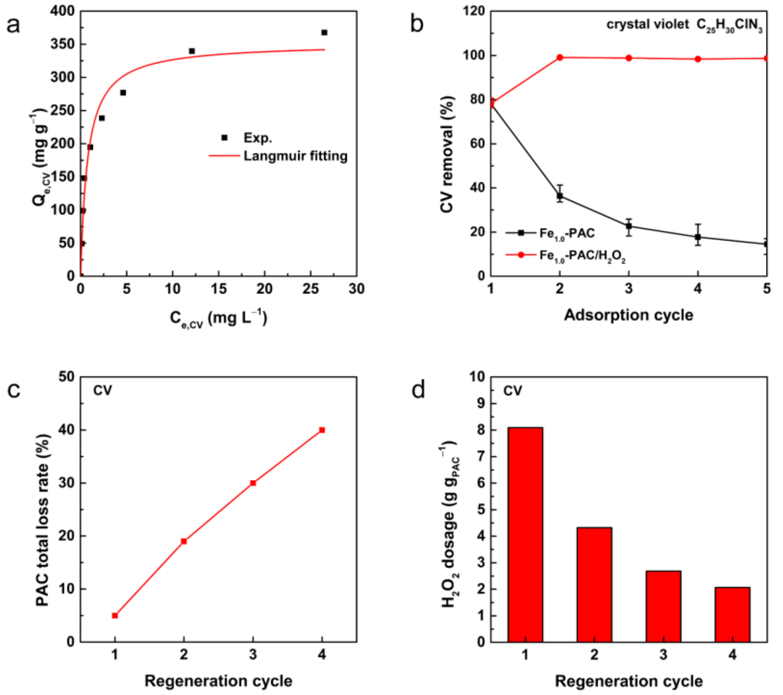

Figure S12. (a) Adsorption isotherm for CV on $Fe_{1.0}$-PAC. (b) Comparison of cyclic CV removal performance in $Fe_{1.0}$-PAC and $Fe_{1.0}$-PAC with H_2O_2 regeneration systems. [CV]=30 ppm (~40% of saturation adsorption), adsorption time=30 min. (c) PAC loss and (d) H_2O_2 dosage during regeneration process for $Fe_{1.0}$-PAC adsorbing CV system.

Note: It is observed that the maximum CV adsorption capacity of $Fe_{1.0}$-PAC (qm) was ~350 mg g^{-1} by the Langmuir isotherm models. The initial removal efficiency of $Fe_{1.0}$-PAC was 78.2% and increased to 98.3% with H_2O_2-regenerationin every cycle, while that of $Fe_{1.0}$-PAC without H_2O_2-regeneration decreased from 78.2% down to 14.5%. The loss rate of $Fe_{1.0}$-PAC was 40.0% and average H_2O_2 consumption was 4.29 g/$g_{(PAC)}$ after 4 regeneration cycles (corresponding to 5 adsorption cycles).

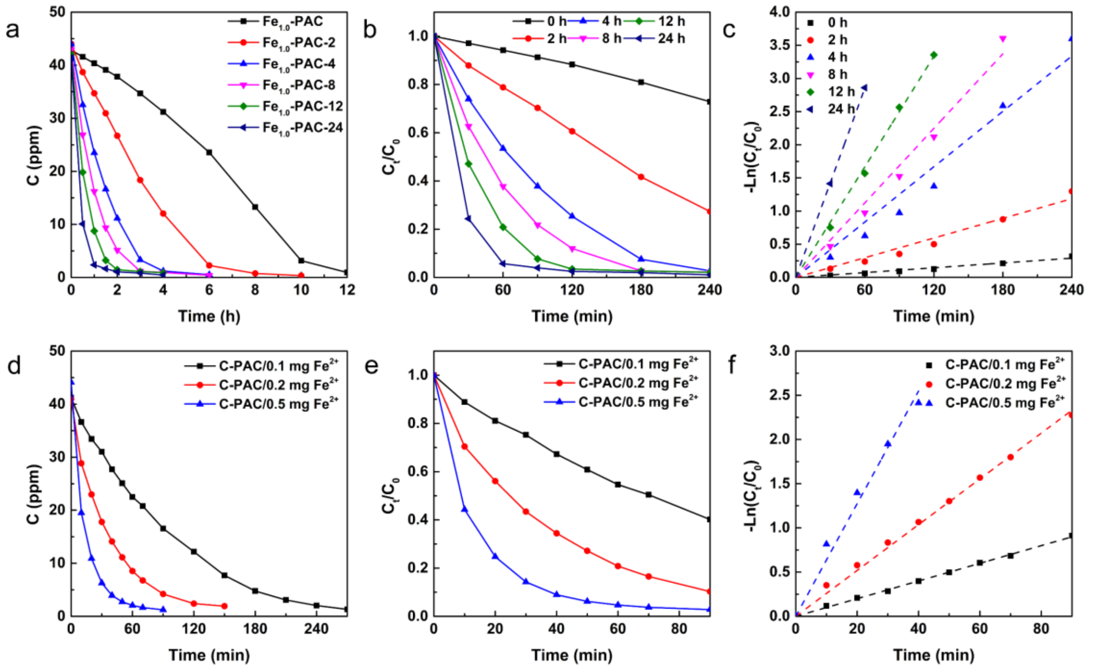

Figure S13. (a) RhB degradation curves of $Fe_{1.0}$-PAC with different activation time. (b) and (c) Langmuir-Hinshelwood quasi-first order kinetic of $Fe_{1.0}$-PAC with different activation time. (d) RhB degradation curves of C-PAC with different amount of Fe^{2+}. (e) and (f) Langmuir-Hinshelwood quasi-first order kinetic of C-PAC with different amount of Fe^{2+}.

Note: The RhB degradation rates and quasi-first order kinetic of $Fe_{1.0}$-PAC increased with the activation time (**Figure S13a-c**). The RhB degradation rates and quasi-first order kinetic of C-PAC/Fe^{2+} systems increased with the addition of Fe^{2+} (**Figure S13d-f**). The *v* and *k* were listed in **Table S1**.

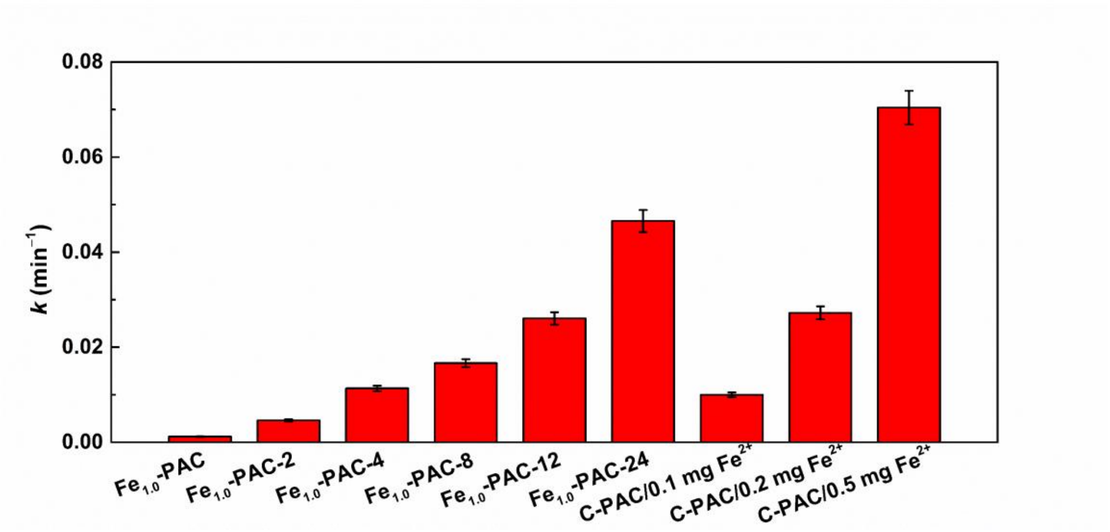

Figure S14. Comparison of pseudo-first-order kinetic constant *k* of $Fe_{1.0}$-PAC with different activation time and C-PAC/Fe^{2+} with different Fe^{2+} content.

Note: The *k*-values were summarized and the *k*-value of C-PAC with 0.5 mg Fe^{2+} was highest (0.0637 min^{-1}) about 1.5-fold of that of $Fe_{1.0}$-PAC-24 (0.0476 min^{-1}).

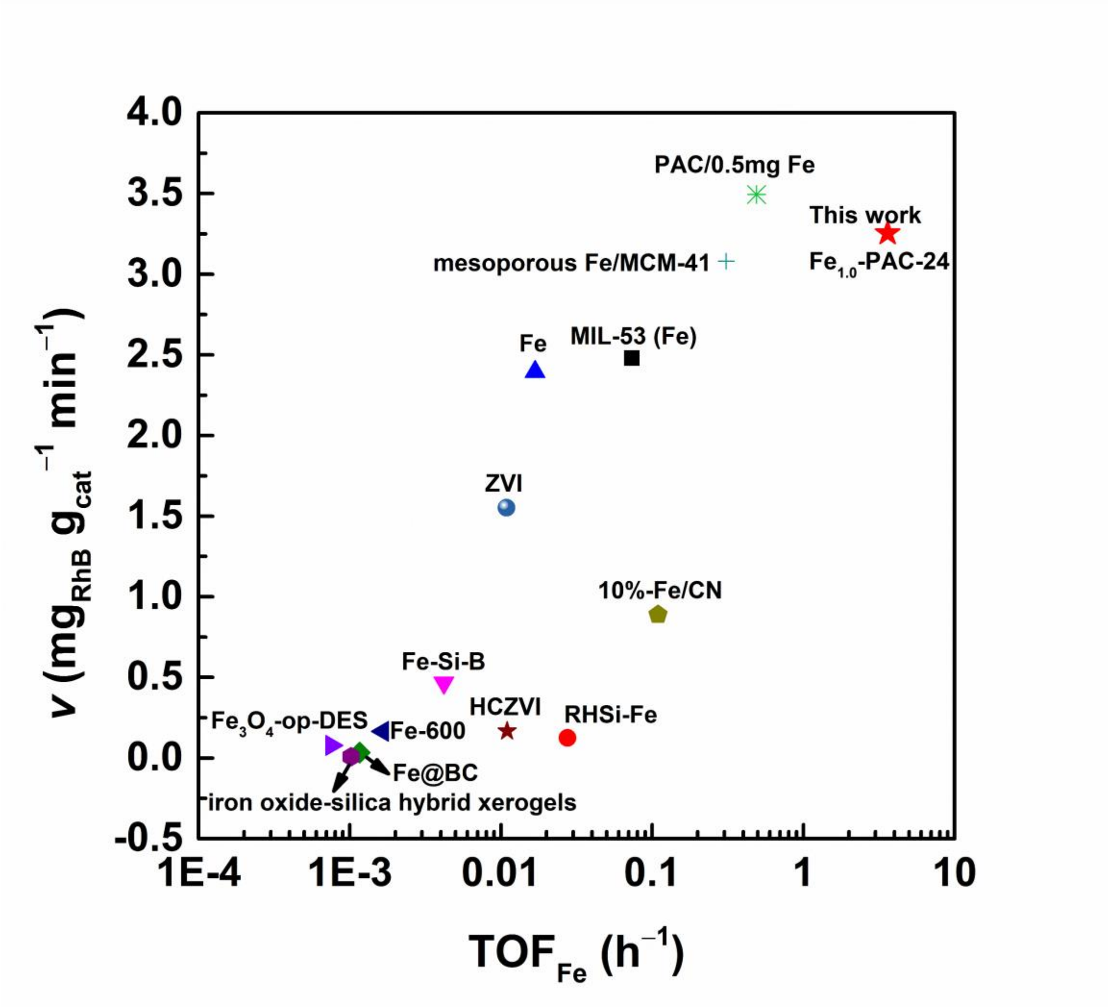

Figure S15. Comparison of mass-based *v*-values and TOF_{Fe} with previously reported Fe-based catalysts activating H_2O_2 for RhB degradation.

Note: The mass-based *v*-value and TOF_{Fe} of $Fe_{1.0}$-PAC-24 were 3.25 mg g^{-1} min^{-1} and 3.61 h^{-1}, and outperformed Fe-based catalysts reported in the literature.

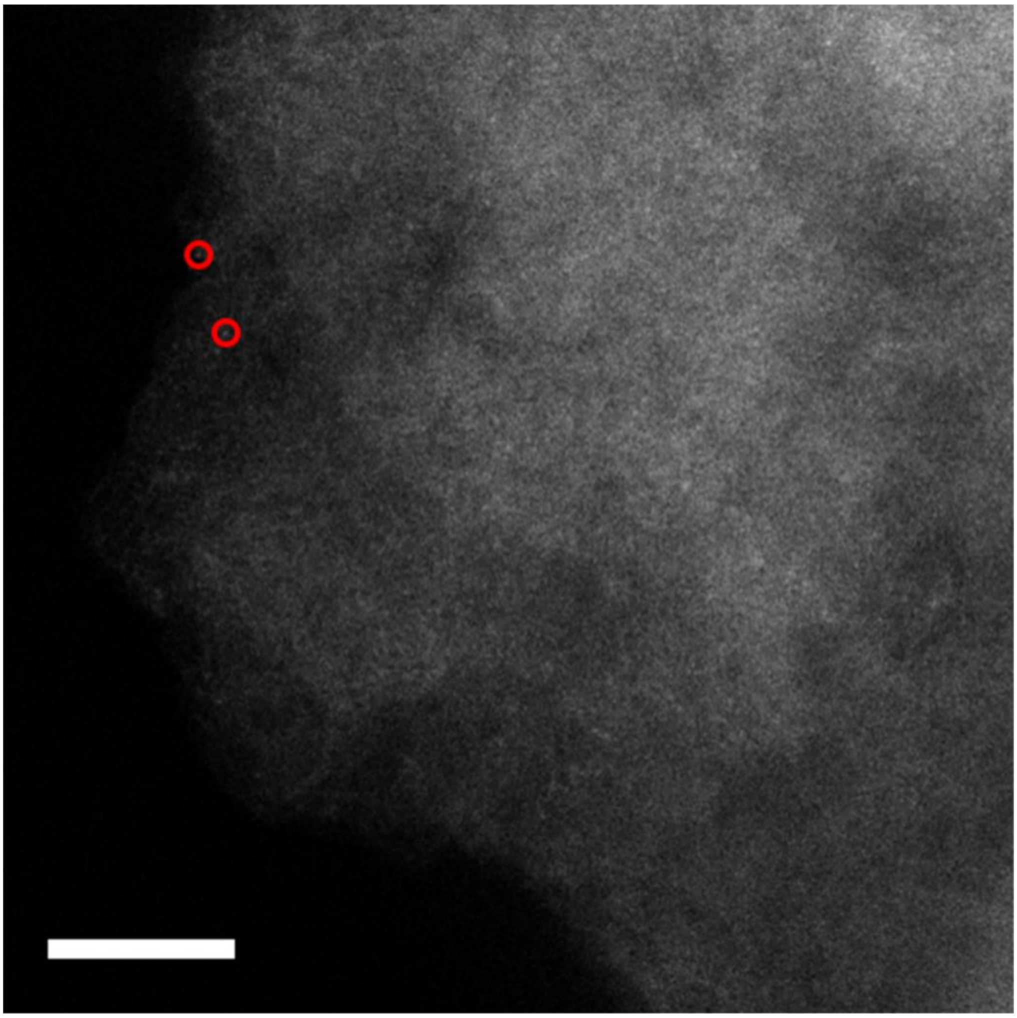

Figure S16. AC-HAADF-STEM image of Fe1.0-PAC-24 after HCl etching. (scale bar 5 nm)

Note: The Fe sites in $Fe_{1.0}$-PAC-24 could be easily etched by HCl solution (almost invisible Fe in AC-HAADF-STEM image)

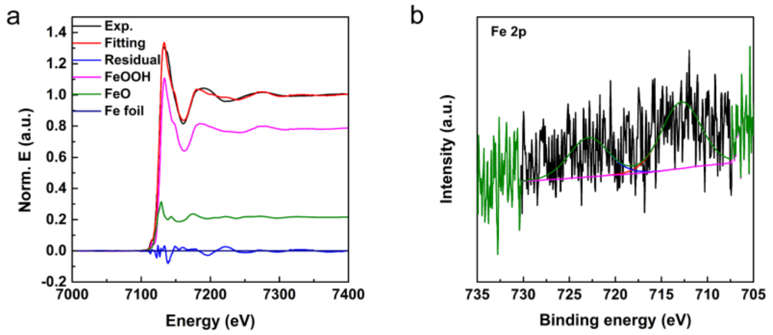

Figure S17. (a) Linear Combination Fitting of valance for $Fe_{1.0}$-PAC-24. (b) XPS spectrum of Fe 2p for $Fe_{1.0}$-PAC-24.

Note: The XAS spectrum of $Fe_{1.0}$-PAC-24 (Figure S16a) was a linear combination of 0.78±0.02 FeOOH (III), 0.22±0.02 FeO (II) and 0.0±0.02 Fe foil (0). The binding energy of Fe $2p_{3/2}$ for $Fe_{1.0}$-PAC-24 was 712.9 eV (Figure S16b), revealing that the valance of Fe was closer to +3.

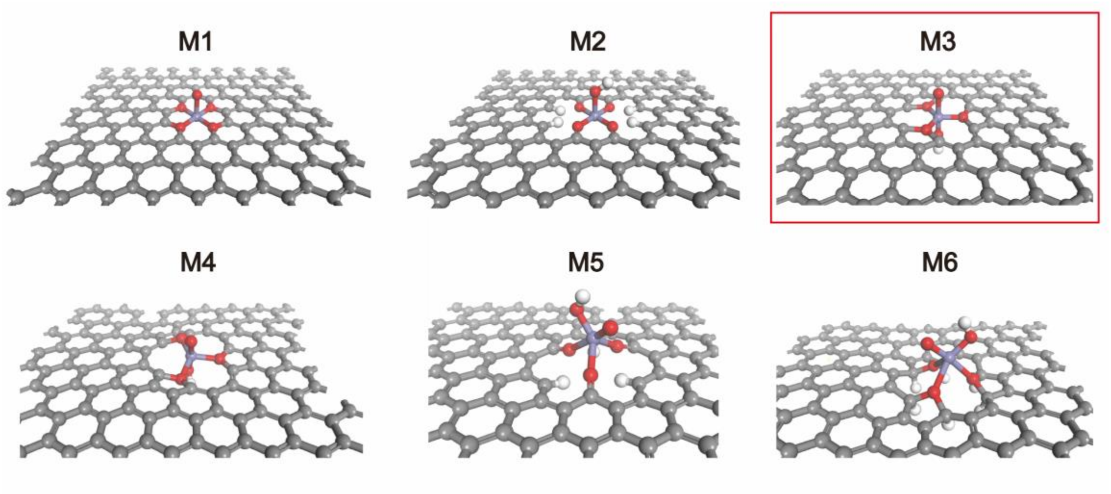

Figure S18. Six possible Fe-doped graphene oxide models.

Note: According to the experiment, we simulated six models, in which Fe is coordinated with 5 oxygen atoms.

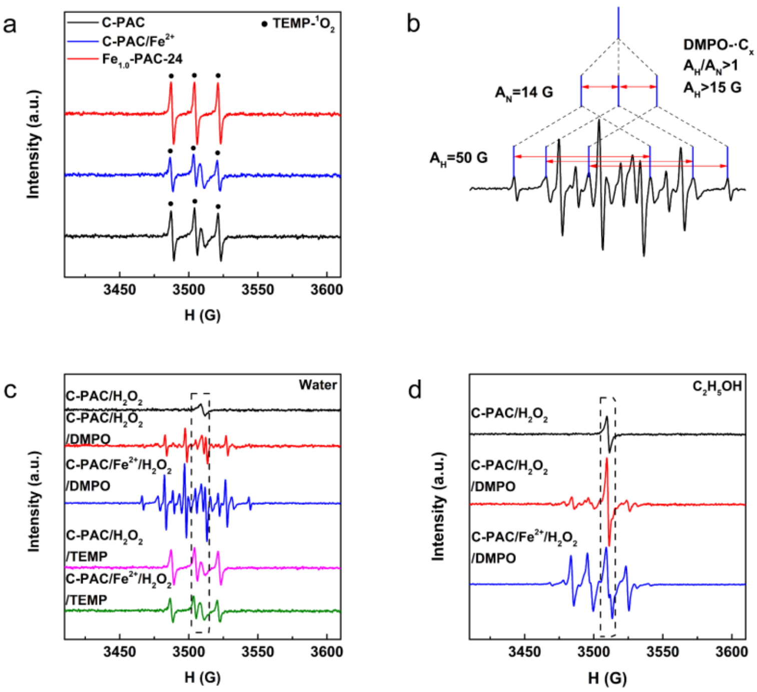

Figure S19. (a) TEMP-trapped EPR spectra of C-PAC/H_2O_2, C-PAC/Fe^{2+}/H_2O_2 and $Fe_{1.0}$-PAC-24/H_2O_2 systems in water. (b) Hyperfine coupling constants of sextet EPR signals in C-PAC/Fe^{2+}/H_2O_2 systems. (c) EPR spectra of C-PAC/H_2O_2, C-PAC/H_2O_2/DMPO, C-PAC/Fe^{2+}/H_2O_2/DMPO and C-PAC/H_2O_2/TEMP, C-PAC/Fe^{2+}/H_2O_2/TEMP systems in water. (d) EPR spectra of C-PAC/H_2O_2, C-PAC/H_2O_2/DMPO C-PAC/Fe^{2+}/H_2O_2/DMPO systems in ethanol.

Note: As **Figure S19a** shown, the triplet signals of TEMP-$^1O_2$ adducts were detected in all of the three systems and the intensities were also similar. The characterized hyperfine coupling constants (HFCs) of sextet EPR signals were A_H = 50 G and A_N = 14 G. A_H/A_N ＞ 1 and A_H ＞ 15 G (**Figure S19b**) indicated that sextet EPR signals belonged to the carbon radicals.[1] The lager HFCs were similar to that of the DMPO-·Si,[2] and C and Si belonged to the same family. Thus, we speculate that sextet EPR

signals might be contributed to the surface carbon radicals of PAC. The peak splitting of C-PAC based systems in water (**Figure S19c**) and signal enhancement of C-PAC based systems in ethanol (**Figure S19d**) were due to the superposition of the signals produced by C-PAC reacting with H_2O_2.

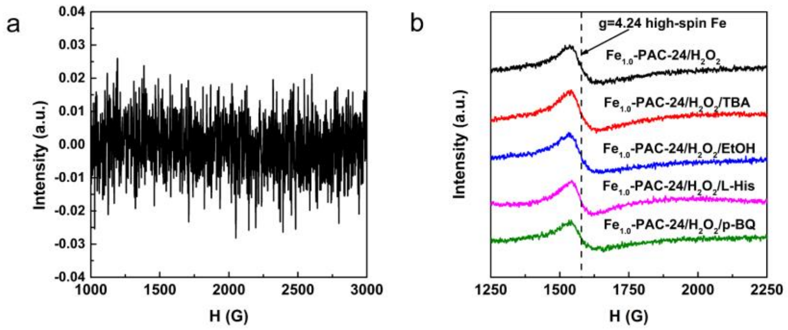

Figure S20. (a) EPR spectra of the quartz tube. (b) EPR spectra of $Fe_{1.0}$-PAC-24/H_2O_2 and with TBA, EtOH, L-His and p-BQ at 100 K.

Note: As shown in Figure S19a, there was no high-spin Fe signal, indicating that the quartz tube did not contain Fe element. The signal of high-spin Fe would not disappear in presence of inhibitors (Figure S19b).

Table S1. The mass-based *v*-value, TOF_{Fe} and *k* of $Fe_{1.0}$-PAC with different activation time and C-PAC with different amount of Fe^{2+}.

Sample	*v* (mg g^{-1} min^{-1})	Fe amount (mg)	TOF_{Fe} (h^{-1})	*k* (min^{-1})
$Fe_{1.0}$-PAC	0.330	0.105	0.220	0.00121
$Fe_{1.0}$-PAC-2	0.580	0.0709	0.574	0.00495
$Fe_{1.0}$-PAC-4	1.13	0.0671	1.18	0.0139
$Fe_{1.0}$-PAC-8	1.16	0.07494	1.09	0.0187
$Fe_{1.0}$-PAC-12	2.15	0.07594	1.99	0.0278
$Fe_{1.0}$-PAC-24	3.25	0.0632	3.61	0.0476
C-PAC/0.1 mg Fe^{2+}	0.907	0.100	0.636	0.00100
C-PAC/0.2 mg Fe^{2+}	1.61	0.200	0.564	0.0259
C-PAC/0.5 mg Fe^{2+}	3.49	0.500	0.490	0.0637

Table S2. Comparison with reported Fe-based catalysts for RhB degradation via H_2O_2 activation.

Catalyst	C_{cat} (g L^{-1})	C_{Fe} (%)	RhB (ppm)	Removal rate (%)	Time (min)	*v* (mg g^{-1} min^{-1})	TOF_{Fe} (h^{-1})
MIL-53(Fe) [3]	0.5	23.7	40	93	30	2.48	0.0734
RHSi-Fe [4]	1	3.18	5	100	40	0.125	0.0276
Fe(0) [5]	1	100	47.9	100	20	2.40	0.0168
Fe-Si-B [6]	0.5	78	20	70	60	0.467	0.00420
Fe@BC [7]	2	20	10	92.7	140	0.0331	0.00116
Fe-600 [8]	1	70	10	99	60	0.165	0.00165
Fe_3O_4-op-DES [9]	0.5	72.4	4.79	98	120	0.0782	0.000758
iron oxide-silica hybrid xerogels [10]	2	5.96	50	100	2880	0.00868	0.00102
HCZVI [11]	0.4	10.66	10	100	150	0.167	0.0110
10%-Fe/CN [12]	0.5	5.7	50	80	90	0.889	0.109
ZVI [13]	0.504	100	47.9	98	60	1.55	0.0109
Fe/MCM-41 [14]	1	7	100	92.4	30	3.08	0.309
$Fe_{1.0}$-PAC-24 (This work)	0.2	0.632	41.4	94.3	60	3.25	3.61
PAC/0.5 mg Fe (This work)	0.2	5	44	95.3	60	3.49	0.490

Table S3. Structural parameters of the $Fe_{1.0}$-PAC-24 catalysts extracted from the EXAFS fitting. (S_0^2=0.76).

Sample	Atomic Scatter	Coordination Number	Bond Length(Å)	Debye-Waller factor(10^{-3}×$Å^2$)	R factor
$Fe_{1.0}$-PAC-24	Fe-O	5.3±0.4	1.99±0.01	6.0±0.8	0.001

Table S4. Summary of the Mössbauer parameters and assignments to different iron species in $Fe_{1.0}$-PAC-24 and $Fe_{1.0}$-PAC-24 with H_2O_2.

Sample	Component	IS (mm s^{-1})	QS (mm s^{-1})	Area (%)	Assignment
$Fe_{1.0}$-PAC-24	D1	0.117	1.844	18.6	HO-($Fe^{II}O_3$)-OOH intermediate-spin
	D2	1.113	2.347	19.7	($Fe^{II}O_3$)=O high-spin
	D3	0.070	0.842	61.7	HO-($Fe^{II}O_3$)=O low-spin
$Fe_{1.0}$-PAC-24 with H_2O_2	D1	0.030	2.072	16.2	HO-($Fe^{II}O_3$)-OOH intermediate-spin
	D2	1.009	2.263	35.9	($Fe^{II}O_3$)=O high-spin
	D3	0.019	0.874	47.9	HO-($Fe^{II}O_3$)=O low-spin

The line width is 0.582 mm s^{-1}.

References

[1] L. Chen, J. Duan, P. Du, W. Sun, B. Lai, W. Liu, *Water Res.* **2022**, 221, 118747.

[2] a) G. R. Buettner, *Free Radical Biology and Medicine* **1987**, 3, 259; b) G. Feng, P. Cheng, W. Yan, M. Borona, X. Li, J. H. Su, J. Wang, Y. Li, A. Corma, R. Xu, J. Yu, *Science* **2016**, 351, 1188.

[3] P. Dinh Du, P. Ngoc Hoai, *Adv. Mater. Sci. Eng.* **2021**, 2021, 5540344, 5540344.

[4] P. P. Gan, S. F. Y. Li, *Chem. Eng. J.* **2013**, 229, 351.

[5] M. F. Hou, L. Liao, W. D. Zhang, X. Y. Tang, H. F. Wan, G. C. Yin, *Chemosphere* **2011**, 83, 1279.

[6] X. Wang, Y. Pan, Z. Zhu, J. Wu, *Chemosphere* **2014**, 117, 638.

[7] C. Wang, R. Sun, R. Huang, *J. Cleaner Prod.* **2021**, 297, 126681, 126681.

[8] S. Guo, Z. Yang, Z. Wen, H. Fida, G. Zhang, J. Chen, *J. Colloid Interface Sci.* **2018**, 532, 441.

[9] F. Chen, S. Xie, X. Huang, X. Qiu, *J. Hazard. Mater.* **2017**, 322, 152.

[10] X. Li, S. Liu, *J. Inorg. Organomet. Polym Mater.* **2021**, 31, 2129.

[11] C. Wang, R. Huang, R. Sun, *J. Mol. Liq.* **2020**, 320, 114421, 114421.

[12] H. A. Bicalho, J. L. Lopez, I. Binatti, P. F. R. Batista, J. D. Ardisson, R. R. Resende, E. Lorençon, *Mol. Catal.* **2017**, 435, 156.

[13] L. Liang, L. Cheng, Y. Zhang, Q. Wang, Q. Wu, Y. Xue, X. Meng, *RSC Adv.* **2020**, 10, 28509.

[14] D. Xu, X. Sun, X. Zhao, L. Huang, Y. Qian, X. Tao, Q. Guo, *Water Air Soil Pollut.* **2018**, 229, 317, 317.
